# The Metabolome of the *Ocotea* spp.: From Biosynthetic Aspects to Bioactive Chemical Scaffolds by Integrating the Genus Chemical Database (*Ocotea*DB)

**DOI:** 10.1021/acs.jnatprod.5c01567

**Published:** 2026-05-19

**Authors:** Albert Katchborian-Neto, Karen de Jesus Nicácio, Paula Carolina Pires Bueno, Miller Santos Ferreira, Matheus Fernandes Alves, Daniele de Oliveira Silva, Wanderleya Toledo dos Santos, Marina de Monroe Gonçalves, Ana Claudia Chagas de Paula, Danielle Ferreira Dias, Marisi G. Soares, RuAngelie Edrada-Ebel, João Henrique G. Lago, Daniela Aparecida Chagas-Paula

**Affiliations:** † Center of Natural Sciences and Humanities, 74347 Federal University of the ABC, Santo Andre, São Paulo 09210-180, Brazil; ‡ Institute of Chemistry, Federal University of Alfenas, Alfenas, Minas Gerais 37130-001, Brazil; § Department of Chemistry, Federal University of São Carlos, São Carlos, São Paulo 14040-901, Brazil; ∥ Department of Chemistry, Federal University of Mato Grosso, Cuiabá, Mato Grosso 14040-901, Brazil; ⊥ Leibniz Institute of Vegetable and Ornamental Crops, Theodor-Echtermeyerweg 1, Großbeeren 14979, Germany; # Institute of Biodiversity, Ecology, and Evolution, Friedrich Schiller University Jena, Dornburgerstraße 159, Jena 07743, Germany; ∇ Department of Pharmaceutical Sciences, 28113 Federal University of Juiz de Fora, Juiz de Fora, Minas Gerais 36036-900, Brazil; ○ Strathclyde Institute of Pharmacy and Biomedical Sciences, 3527 University of Strathclyde, Glasgow G4 0RE, Scotland; ◆ Department of Chemistry, Federal University of Juiz de Fora, Juiz de Fora, Minas Gerais 36036-900, Brazil

## Abstract

The genus *Ocotea* is a significant source of bioactive agents within the Lauraceae, yet it remains underexplored. Despite its ethnomedicinal relevance and chemical diversity, *Ocotea* species remain taxonomically challenging as they belong to the multifaceted *“Ocotea* complex”, a phylogenetically unresolved group. Moving beyond previous literature *Ocotea* surveys, this review provides the first curated, genus-specific data set of both volatile and nonvolatile specialized metabolites. Covering research articles from 1830 to 2025, this review provides the most comprehensive synthesis to date, documenting 984 unique chemical compounds across 115 species. This effort culminated in the construction of the *Ocotea* Chemical Database (*Ocotea*DB), a novel digital resource created to streamline the genus’s chemical diversity for the global scientific community. Furthermore, it presents current knowledge of biosynthetic routes leading to key bioactive scaffolds, including aporphinoid and benzylisoquinoline alkaloids, lignoids, glycosylated flavonoids, and a diverse array of terpenoids, while addressing the stereochemical and structural intricacies unique to the genus. This review lays a robust foundation to drive future *Ocotea*-focused bioprospecting studies and guides research in the fields of natural products, chemophenetics, metabolomics, and medicinal chemistry regarding the *Ocotea* species, its chemical scaffolds, and specialized metabolites.

## Graphical abstract

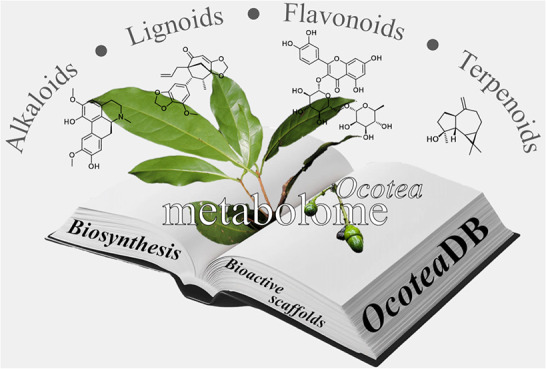

## Introduction

The *Ocotea* Aubl. genus belongs to the Lauraceae Jussieu, popularly known as the laurel family, which comprises more than 3,000 species distributed among 55 genera worldwide.^1,2^ The Lauraceae is among the five most abundant families in tropical and subtropical forests worldwide, classified into the Magnoliid clade as part of the order Laurales.^3–6^ Several Lauraceae plants have traditional culinary uses—for example, laurel (*Laurus nobilis*) is used as a flavoring agent to season food, and cinnamon (*Cinnamomum verum*) is an important spice. *Persea americana* is a tree that produces one of the most important tropical fruits in the world, popularly known as avocado.^4–8^ In general, Lauraceae species comprise aromatic trees, several of which are recognized as natural sources of essential oils. Examples include *Aniba rosaeodora*, known in Brazil as “pau-rosa”, and *Ocotea catharinensis*, known as “canela-preta”, both well-known for their high content of linalool, a highly sought-after monoterpenoid for enhancing woody fragrances in several commercial products.^6,9–11^

*Ocotea* is the largest genus of the Lauraceae, comprising more than 400 species,^1^ and several species have been documented as popular medicines, known for their significant antioxidant, analgesic, anti-inflammatory, and antitumor properties.^4,5,12^ *O. quixos*, for example, is traditionally used as an anesthetic for wounds, skin and stomach aches.^13,14^ Other species, such as *O. porosa*, *O. odorifera*, and *O. catharinensis*, are known as a source of woody of high-quality desirable timber, which have been used in carpentry and civil constructions for decades in South America.^6,15^

Phytochemically, *Ocotea* spp. species are frequently composed of alkaloids, lignoids, flavonoids, and terpenoids.^4^ To date, only 115 species (∼28.5%) have been investigated for their chemical content in classical or modern Natural Products (NP) studies, including our recent comprehensive metabolic profiling study (Supplementary Tables S1, S1.1, and S1.2).^16^ The majority of *Ocotea* species are neotropical, found mainly in Central and South America, though some species are also found in Western and Southern Africa, and in Macaronesia, Madagascar, and the Comoro Islands.^1,14,17^ In South America, the genus *Ocotea* is the third most common in the Amazon biome, which offers the largest biodiversity hotspot in the world. In the main Brazilian biomes (Amazon Rainforest, Atlantic Rainforest, Cerrado, Caatinga, and Pampas), a total of 176 species have been registered, of which 112 are considered endemic.^18^ It is estimated that more than 150 species remain to be located, taxonomically identified, and phytochemically investigated.^19,20^

Botanically, *Ocotea* is considered an intriguing genus that is, at present, classified as belonging to the *Persea*-*Laurea* clade. Phylogenetically and based on DNA sequencing markers and morphological features, *Ocotea* belongs to the *Ocotea* complex *sensu*, which currently includes approximately 950 species of 17 distinct genera.^21,22^ The *Ocotea* complex is of significant interest to NP chemists due to diverse biological activities, such as anti-inflammatory, antibacterial, antiviral, and insecticidal properties.^4^ However, when DNA material is unavailable for comparison, the taxonomic identification of *Ocotea* species based on only morphological characteristics is challenging, and these can be misclassified as species from other genera of the *Ocotea* complex, such as *Nectandra*, *Persea*, *Licaria*, and *Litsea*. Thus, *Ocotea* is considered one of the most challenging genera for species identification within the Lauraceae family.^1,19,21,23,24^ Plant systematics, including chemical and molecular aspects, aids in the taxonomic classification of *Ocotea*.^2,8,22^ However, despite significant ethnomedicinal, ecological, and economic relevance of this genus, the chemical space of many species is still uncharacterized. Besides, unfortunately, several *Ocotea* spp. are currently threatened with extinction, highlighting the importance of efforts to protect them.^15^

### Biosynthesis, Chemical Aspects, and Bioactivities

The first academic medical report on *Ocotea* was published in 1830, noting the therapeutically valuable oil of an *Ocotea* tree native to South America. Various parts of the tree were used to relieve spasmodic complaints, convulsions, and cramps, while effective against acute and chronic inflammation in the treatment of cutaneous eruptions or rheumatic pain was also reported.^25^ Data regarding the chemical aspects of *Ocotea* oil were first published in 1844, along with the coinage of the term “oil of laurel turpentine” in the literature.^26^ Likewise, the first nonvolatile compounds reported for *Ocotea* were lignans, including sesamin (pseudocubebin), isolated in 1916 from the bark of *O. usambarensis*, a traditional medicinal plant used by African natives in the Kimboza Forest Reserve of Tanzania.^27,28^

In the past 70 years, and notably in the current 21st century, a large number of scientific papers have demonstrated that *Ocotea* is a promising bioactive genus with relevant antimicrobial, larvicidal, anesthetic, anti-inflammatory, and antitumor properties.^4,19,29–32^ Concerning its phytochemistry, the genus *Ocotea* is perceived as significantly diverse, with a high degree of chemical diversity along with its specialized metabolites. Alkaloids are the most frequently occurring chemical class in the genus, and a series of different subclasses has been observed, including a wide range of aporphines and benzylisoquinolines and the less common morphinans and phenanthrenes.^4,19,33,34^ The most shared alkaloid classes in the *Ocotea* genus consist of isoquinoline derivatives, with aporphines making up the majority (Figure 1).

**Figure 1:**
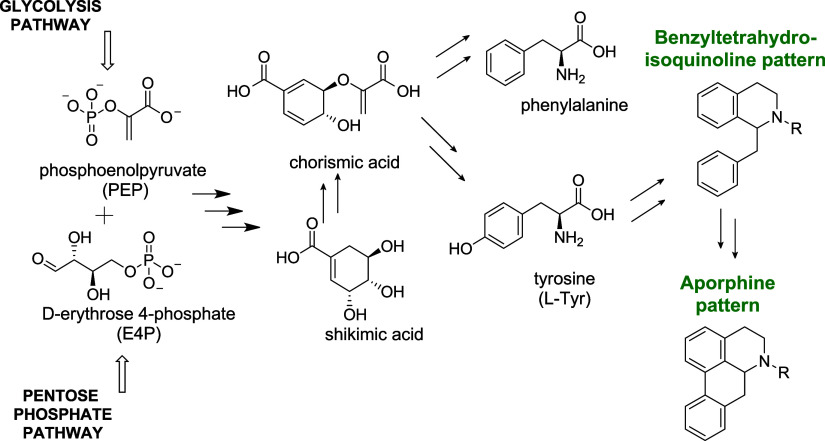
Schematic shikimate pathway: PEP and E4P → shikimic acid → chorismic acid → aromatic amino acids → tyrosine, which yield benzyltetrahydroisoquinoline and aporphine alkaloids scaffolds.

Aporphine alkaloids serve as key chemotaxonomic markers of the genus and constitute one of the major compounds of anti-inflammatory, antimicrobial, and antiparasitic activities.^31,35–37^ Succeeding the alkaloids, the lignoids and flavonoids are the most commonly described NP in the *Ocotea* genus, which will be covered in this review. The metabolome of *Ocotea* also includes volatile alkylphenols, aldehydes, terpenoids, phenylpropanoids and other less common metabolites such as coumarins, tannins, butenolides, saponins, glycosides, benzopyrans, and steroids (Supplementary Tables S2–S7).^4,19,38–41^

More recently, using modern analytical molecular methods, several metabolites could be chemically discriminated in a given taxon—a chemophenetic approach, which can be considered a modern extension of chemotaxonomy.^42^ Metabolomics associated with DNA sequencing can also aid in solving phylogenetic issues. In addition, metabolic fingerprints can enable the identification of chemical markers within the *Ocotea* genus—for example, metabolites from the classes of morphinans and *O*-glycosylated flavonoids can be used for metabolic fingerprint discrimination within the genus. The flavone:flavonol ratio can also assist in evolutionary lineage pattern studies, once they are linked to the species specialization index, and their chemical presence can be associated with genetic modifications during the evolution of these plants.^19^ A recent application of that includes the use of the metabolic fingerprint content to avoid taxonomic errors in laboratories and industry arising from morphologically similar botanical features.^43^ In particular, metabolomics can also support a more rapid discovery of known or unknown bioactive metabolites via high-throughput screenings.^44–48^

Thus, a robust chemical database associated with the main biosynthetic pathways of the *Ocotea* metabolome can facilitate the recognition of new potential biomarkers and achieve more accurate and faster species differentiation. Herein, we present and discuss the most comprehensive chemical review of the *Ocotea* genus. The main objective is to provide an updated version of current knowledge on the chemical and biosynthetic characteristics of the *Ocotea* metabolome to offer additional insights and orient further studies targeting *Ocotea* species, culminating in the creation of the *Ocotea* chemical database (*Ocotea*DB). It is also intended to provide easy access to chemical cores and unique metabolites, integrated with main biosynthetic routes to access their chemophenetic value.

### *Ocotea* spp. Specialized Metabolites

Alkaloids, lignoids, and terpenoids are the main representative classes found, and flavonoids are also found, though in a smaller number of *Ocotea* spp.^4,8,16,49^ Despite notable discoveries by Professor Otto R. Gottlieb (University of São Paulo, deceased in 2011) and collaborators between 1960 and 1980, research interest has only recently shifted back to the genus, with an increased number of relevant publications in the past 15 years.^19,22,50^ In this review, a total of 984 metabolites were compiled into the *Ocotea*DB, based on data retrieved from both the scientific literature (1830 and 2025) and NP databases, including: Web of Science, ScienceDirect, Google Scholar, Dictionary of Natural Products (DNP^©^), KNApSAcK,^51^ and the Nuclei of Bioassays, Biosynthesis and Ecophysiology of Natural Products Database (NuBBE).^52^ Inclusion criteria were limited to peer-reviewed studies identifying volatile and nonvolatile *Ocotea* compounds via Nuclear Magnetic Resonance (NMR) and liquid chromatography coupled to mass spectrometry (LC-MS), or gas chromatography-MS (GC-MS). Duplicates were manually checked and filtered, while inconsistencies in chemical nomenclature were resolved using the IUPAC standard and cross-references with PubChem, ChemSpider and SciFinder. The *Ocotea*DB includes detailed information such as chemical and/or trivial names, molecular formulas, monoisotopic masses, metabolite scaffold, biosynthetic classes, SMILES, *Ocotea* species where it was identified, and respective bibliographic references (see Supplementary Tables S2–S7).

### Alkaloids

The main alkaloid class produced by *Ocotea* comprises the aporphines. This class is not exclusive to Lauraceae, as it also occurs in other plant families, such as Magnoliaceae, Menispermaceae, Papaveraceae, Ranunculaceae, Hernandiaceae, and Annonaceae.^53,54^ Aporphine alkaloids are one of the largest groups of isoquinoline alkaloid derivatives, with more than 500 known representatives in the literature. Despite the chemical diversity and distribution of this class, robust data regarding its metabolic biosynthetic pathways in the literature are sparse.^49,55^

The biosynthesis of the aporphine core includes a Pictet–Spengler reaction type, where a Mannich-like condensation takes place to join dopamine and 4-hydroxyphenylacetaldehyde precursors by amino alkylation and form (*S*)-norcoclaurine (**1**), the primary precursor for the benzylisoquinoline class of compounds. *O*-methylation of **1,** yielding (*S*)-coclaurine (**2**), is mediated by norcoclaurine 6-*O*-methyltransferase (E1) and *S*-adenosyl methionine (SAM), common substrates involved in the stereoselective mechanism of methyl group transfers via a S_N_2-type reaction. Subsequently, precursor **2** is catalytically converted into (*S*)-*N*-methylcoclaurine (**3**) by coclaurine *N*-methyltransferase (E2). Following the pathway, alkaloid **3** can be hydroxylated at position 3′ of the benzylic ring to form the tetrahydroxy-substituted pattern in the presence of the enzyme *N*-methylcoclaurine 3′-hydroxylase (E3), to afford (*S*)-3′-hydroxy-*N*-methylcoclaurine (**4**). In addition, the hydroxyl group at position 4′ can be methylated by 3′-hydroxy-*N*-methylcoclaurine-4′-*O*-methyltransferase (E4)/SAM to afford the alkaloid (*S*)-reticuline (**5**), which is one of the main benzylisoquinoline alkaloids found in the *Ocotea* genus. For instance, alkaloid **5** has been isolated from several species, including *O. lancifolia*, *O. caparrapi*, *O. odorifera*, and *O. caudata*, among others.^4,34,49^ The biosynthetic pathway is depicted in Figure 2.

**Figure 2:**
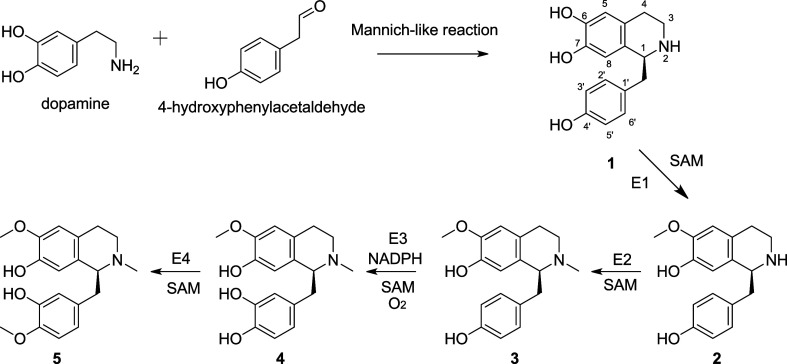
Biosynthetic pathway of benzylisoquinoline alkaloids **1**–**5**. E1-norcoclaurine 6-*O*-methyltransferase; E2-coclaurine *N*-methyltransferase; E3-*N*-methylcoclaurine 3′-hydroxylase; E4-3′-hydroxy-*N*-methylcoclaurine-4′-*O*-methyltransferase.

Although *Ocotea* does produce *R* isomers as well,^53^ the dominant presence of *S* isomers in the *Ocotea* genus has been described for benzyltetraisoquinoline alkaloids. The biosynthesis of either the *R* or *S* stereoisomers after the Mannich reaction is expected to be dependent on the configuration of the precursor. For instance, previous studies reported (6*R*)-reticuline (**8**) from *O. diospyrifolia*, while (6*S*)-reticuline has been obtained from *O. odorifera*.^56,57^ In addition, different (*R*)-alkaloid isomers have been reported from a few other *Ocotea* species, such as *O. caesia*, *O. lancifolia*, and *O. velloziana*.^37,49,58^ These findings corroborate the significance of chemophenetics and biosynthetic studies, which, to date, are poorly described in the literature. This area can, therefore, be further explored by using specific enzymes and substances with atom labeling to confirm the assumptions and observations highlighted in this review.^59^ Furthermore, alkaloid **5** can undergo phenolic oxidative coupling, which plays a significant role in modifying the basic benzyltetrahydroisoquinoline skeleton to yield several other types of alkaloids, such as aporphines and morphinan derivatives. While the former is widespread in the *Ocotea* genus, the latter is also present, though in fewer *Ocotea* species.^33,53,60^ The aporphines (*S*)-corytuberine (**6**) and (*S*)-isoboldine (**7**) have been identified in different *Ocotea* species, such as *O. caesia*, *O. caudata*, *O. lancifolia*, and other Lauraceae genera.^4,19,33^ Literature data^53,61^ support the change in the absolute configuration of **5** to the *R* enantiomeric form (**8**), which can be achieved by an oxidation–reduction process via the formation of the 1,2-dehydroreticulinium cation intermediate (**9**). The *R* enantiomer is produced via oxidation, which turns it up in a planar iminium cation, enabling the formation of **8**. These steps allow for the stereochemical change through the action of two coordinated enzymes: 1,2-dehydroreticuline synthase (E5)/NADP^+^, which is responsible for the first heterocyclic nitrogen oxidation, and 1,2-dehydroreticuline reductase (E6)/NADPH, reducing the ion to form the enantiomeric alkaloid **8**. The classes of morphinans found in *Ocotea* are predominantly biosynthetically derived from the (*R*)-reticuline (**8**) stereoisomer.^53,61,62^ The *Ocotea* spp. are not known to be morphinan producers as species from Papaveraceae; however, methylated morphinans such as pallidine (**9**) have already been isolated from *O. acutangula*, *O. acutifolia*, and *O. lancifolia*.^61^ Curiously, the most known morphinans, such as codeine, salutaridine, and morphine, have not yet been found in *Ocotea* spp. Biosynthetically, morphinan alkaloids are produced by reactions mediated by coupling enzyme synthases that are cytochrome P450-dependent monooxygenases, such as salutaridine synthase (E7), responsible for giving rise to the dienone morphinan alkaloids (Figure 3).

**Figure 3:**
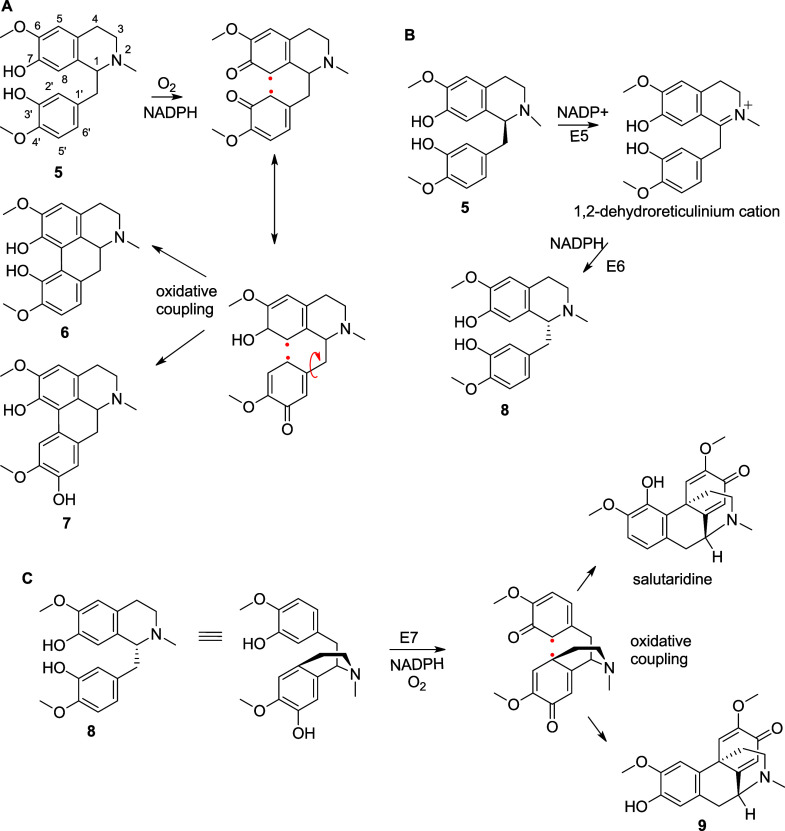
Biosynthetic pathway of aporphine, morphinan alkaloids **6**–**9** and related compounds from (*S*)-reticuline (**5**). (A) Oxidative coupling of (S)-reticuline to forming aporphine alkaloids; (B) isomerization of (*S*)-reticuline (5) to (*R*)-reticuline (8); (C) Conversion of (*R*)-reticuline (8) into salutaridine and morphinan derivatives. E5-1,2-dehydroreticuline synthase; E6-1,2-dehydroreticuline reductase; E7 - salutaridine synthase.

Moreover, the CYP complex enzyme CYP80A1 catalyze the C–O intermolecular phenol-coupling reaction between benzylisoquinoline cores to afford a dimer class of alkaloids, the bisbenzylisoquinolines. Bisbenzylisoquinolines occur in plant families such as Lauraceae, Menispermaceae, Berberidaceae, and Ranunculaceae, but they are not characteristic of *Ocotea* spp., where they are rarely reported. Within *Ocotea*, the bisbenzylisoquinolines, tetrandrine and thalmine have only been described in two species to date, *O. rodiei* and *O. venenosa*, both of which were historically assigned to the genus, but later reclassified as *Chlorocardium rodiei* and *C. venenosum* based on morphological and phylogenetic evidence.^4,19,63^

### Alkaloid Profile

In their comprehensive review using multivariate statistical data analysis, Antonio et al. (2020) claimed that approximately less than 10% of *Ocotea* species have been chemically recognized for their alkaloid content.^19^ Their study employed 31 alkaloid-producing *Ocotea* species, as extensively described earlier by Teles et al. (2019).^49^ The present review has updated this number to 60 *Ocotea* species reported to date for eliciting biosynthetic pathways for alkaloid production.^16^ Thus, the alkaloid class stands as the most prevalent class among the investigated *Ocotea* species, with 156 curated reported and well described metabolites (Supplementary Tables S2 and S2.1). Regarding only classical phytochemistry studies, aporphine alkaloids constitute the most common alkaloid source isolated from *Ocotea* spp., with 107 unique representative structures in the literature (Figure 4).

**Figure 4:**
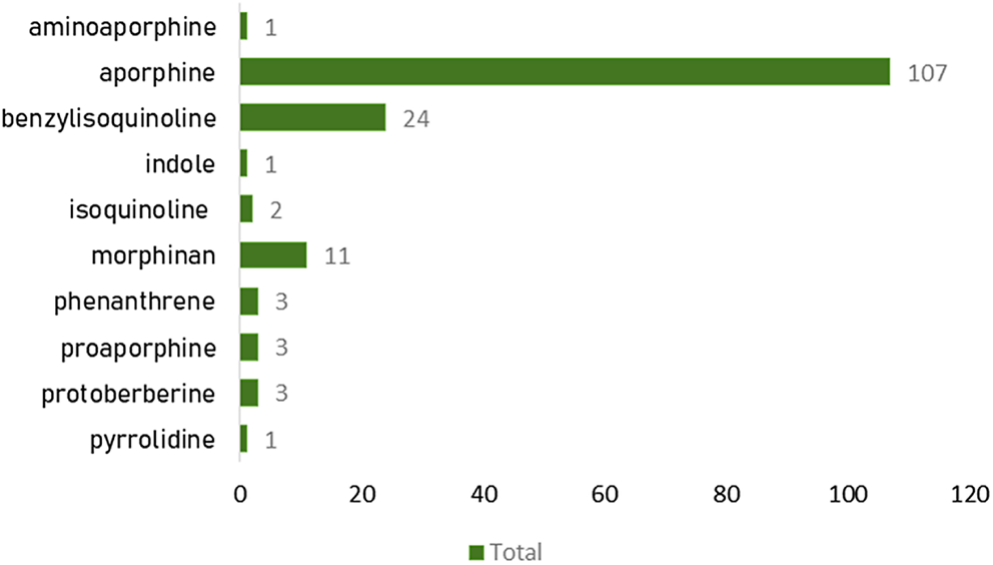
Chemical diversity of alkaloids in the *Ocotea* genus is expressed as the number of different chemical structures reported in the literature for each subclass.

From the 60 alkaloid-producing species, 55 (∼91.5%) had the aporphine core described in at least one study (core **C2**). Moreover, beyond the aporphines, classes such as their precursors or derivatives that include benzylisoquinoline, proaporphine, phenanthrene, morphinans, and protoberberines have been reported in a fewer number of *Ocotea* species, with a total of 43 unique metabolites (cores **C1** and **C3–C6**). Even less common chemical cores have been reported; for example, aminoaporphine (core **C7**) has been isolated from *O. variabilis*, the indole core (**C8**) from *O. minarum*, the pyrrolidine core (**C9**) from *O. caudata*, and the isoquinoline core (**C10**) from *O. diospyrifolia*.^4,57,64,65^ Additionally, the isoquinoline core has been recently annotated in the *O. delicata*.^66^ The different alkaloid chemical scaffolds found in *Ocotea* species are displayed in Figure 5.

**Figure 5:**
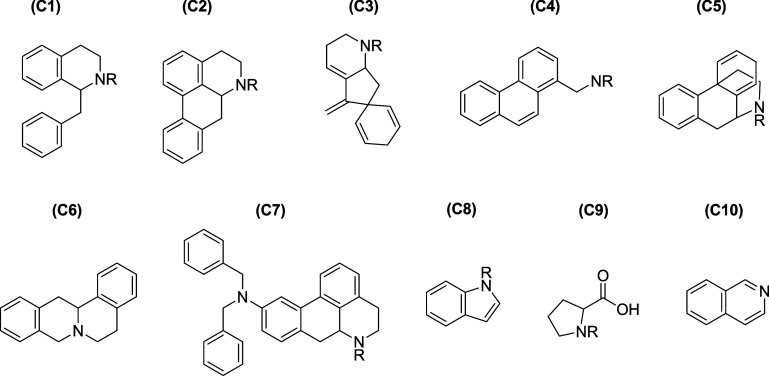
Alkaloid chemical scaffolds found in *Ocotea* species: (**C1**) benzylisoquinoline, (**C2**) aporphine, (**C3**) proaporphine, (**C4**) phenanthrene, (**C5**) morphinan, (**C6**) protoberberine, (C7*) aminoaporphine, (C8*) indole, (C9*) pyrrolidine, and (C10*) isoquinoline alkaloids from the *Ocotea* genus. *Only one study in the literature has reported the isolation of the chemical core. Note: depending on each class scaffold, R = H/H_2_/O^–^/CH_3_.

Aporphines are a relevant alkaloid class for evolutionary lineages in Lauraceae and are considered a biomarker for discriminating between *Ocotea* as a basal genus and other genera in the family.^19^ For example, the patterns of the substituted aporphines can aid in the differentiation of *Ocotea* from *Cinnamomum* and even other genera placed at the unresolved *Ocotea* complex *sensu*.^1,19^ Thus, an alkaloid fingerprint profile including aporphine patterns can support phylogenetic studies of Lauraceae, providing significant key information to elucidate alkaloid expression, complementary to gene sequencing. There are different subclasses of aporphine in *Ocotea* species, such as dihydroaporphines, didehydroaporphines, oxoaporphines and didehydroxoaporphines. However, within the *Ocotea* genus, only specific substituted aporphines can serve as significant differential biomarkers, especially those with high levels of oxidation (e.g., 3-hydroxydicentrine or dehydroocoteine).^19^ This is expected, as the majority of *Ocotea* species are aporphine producers and share the same biosynthetic pathway. Thus, besides the potential for genus differentiation within the *Ocotea* complex, other metabolite classes should be utilized as a more efficient biomarker selection for a chemosystematics of the *Ocotea* genus.

Phylogenetic and evolutionary classification states that *Ocotea* species producing majoritarian aporphines are part of the Old World, and thus, it is assumed that they are more basal in the Lauraceae lineage.^1,19^ On the other hand, *Ocotea* species that produce more evolved alkaloid cores, such as the morphinan, could be considered to be from the New World, as they have evolved from specialized biosynthetic pathways; for example, in *O. acutangula*, where six different morphinan alkaloids have been isolated. Only five *Ocotea* species (*O. acutangula*, *O. acutifolia*, *O. brachybotrya*, *O. caudata*, and *O. lancifolia*) have been described in the literature as morphinan producers.^1,4,30,34,49,64^

Furthermore, chemometrics and metabolomics studies can aid in the search for specific biomarkers and alkaloidal fingerprints to facilitate species differentiation. For example, plants from the *Licaria* and *Nectandra* genera might be taxonomically confused with those from the *Ocotea* genus due to their botanical similarity in terms of leaf and flower morphology.^21^ Some of these species can share the same stomatal surface shape, making morphological differentiation difficult. Thus, plants such as those from the *Ocotea* genus can be more easily differentiated by their chemical profiles and through biomarker comparisons when accompanied by chemometrics analyses of their specialized metabolites. Chemical profile data, together with respective morphological features and phylogenetic studies, can avoid inaccurate taxonomic assumptions and incorrect genus identifications.^19,67^

Regarding its chemistry and bioactivity, aporphine cores exhibit diverse chemical possibilities in terms of substituents, including methoxy, methylenedioxy, and hydroxy substituents attached at different positions to the aromatic ring.^33,34,60^ This review describes all substituted aporphine cores within the *Ocotea* genus reported in the literature, including the derivative classes of oxoaporphines, proaporphines, *O*-aporphines, dihydroaporphines, didehydroaporphines, and phenanthrenes (Figures 4 and 5). The detailed identifications of the chemical compounds, including their monoisotopic masses, molecular formulas, chemical names, SMILES, and *Ocotea* species from which the metabolites were isolated (Supporting Information file, Tables S2–S7).

Furthermore, aporphine alkaloids (**C2**) from different *Ocotea* species have been reported with pronounced anti-inflammatory and antineoplastic properties. Examples include the aporphine boldine, the most prevalent scaffold found in *O. lancifolia*, *O. spixiana*, and other *Ocotea* spp. and Lauraceae species, which can induce antipyretic effects possibly due to the effect on the COX pathway, leading to the inhibition of prostaglandin’s release.^68,69^ Likewise, the aporphine alkaloid glaucine, which occurs in *O. vellosiana* and *O. macrophylla*, exhibits interesting anticancer properties by suppressing the activity of nuclear factor kappa-activated B cells (NF-κB). In addition, the glaucine congener has also demonstrated a reduction in metastatic breast cancer cell invasion.^70^ Recently, nuciferine-derived aporphine alkaloids were associated with *O. villosa* cytotoxicity against MCF-7 human breast cancer cells.^71^

The aporphine alkaloid dicentrine and the oxoaporphine dicentrinone have demonstrated a broad spectrum of biological activities, such as the inhibition of topoisomerase I and II enzymes, which are relevant therapeutic targets of current chemotherapy protocols. In addition, more recent data have confirmed the significant *in vivo* antinociceptive effects of dicentrine. The mechanism of action includes attenuating mechanical and cold hypersensitivity under inflammatory conditions via the activation of the transient receptor potential TRPA1.^72^ Dicentrinone has also shown potent antiparasitic activity against the trypomastigote forms of *Trypanosoma cruzi* and significant changes in lipid biological surfaces with reduced mammalian cytotoxicity.^36,73^ Moreover, glaziovine, a rare proaporphine isolated from *O. glaziovii*, exhibit remarked anxiolytic effects and was indeed registered as a tranquilizer under the trademark Suavedol by the pharmaceutical Simes S.p.A., Milano.^74–76^

Regarding the benzylisoquinoline alkaloids (**C1**) (Figure 5), *R*-coclaurine has demonstrated a highly pronounced anti-HIV activity, with an EC_50_ value of 0.8 μg/mL.^77,78^ Furthermore, promising butyrylcholinesterase inhibition activities have been reported for two other benzylisoquinoline alkaloids: (+)-reticuline, with an IC_50_ value of 33.6 ± 3.0 μM, and (+)-*N*-methylcoclaurine, with an IC_50_ value of 15.0 ± 1.4 μM.^79^ The *O. odorifera* leaf decoction is rich in alkaloid (+)-reticuline, which, after alkaloid isolation and evaluation, exhibited a significant antiedematogenic effect together with neutrophil recruitment inhibition in a dose-dependent manner, suggesting that both the COX and LOX inflammatory pathways were inhibited.^56,80^ Benzylisoquinolines and aporphines are the major classes isolated from *Ocotea* species and can be considered major bioactive NP in the genus. They are encountered in several different *Ocotea* species, such as *O. puberula*, *O. vellosiana*, *O. acutifolia*, *O. macropoda*, *O. leucoxylon*, *O. discolor*, *O. caesia*, *O. odorifera*, *O. diospyrifolia*, *O. lancifolia*, and *O. brachybotrya*, among others (Supplementary Tables S2 and S2.1). Besides, other members of the broader benzylisoquinoline/isoquinoline alkaloid space have reached the pharmaceutical market, even if they were not isolated from *Ocotea* spp. In particular, apomorphine, an aporphine alkaloid, is an established drug for Parkinson’s disease,^81^ and papaverine, a benzylisoquinoline alkaloid, has long been used clinically as a vasodilator/antispasmodic.^82^

### *Ocotea* spp. Lignoids

Since the identification of the highly bioactive lignan podophyllotoxin in 1933, the biosynthetic pathways of lignoids have been thoroughly elucidated, enabling a clear understanding of their core bioactive scaffolds, such as the podophyllotoxin 8–8′, 2–7′ aryltetralin lactone derivatives. Although some gaps persist in the literature regarding the most specific biosynthetic steps, particularly for the more complex neolignans, which exhibit vast structural and stereochemical diversity.^39,83,84^ Given their pharmacological relevance, podophyllotoxin and related aryltetralin lignans have become central targets of synthetic, biosynthetic, and medicinal-chemistry investigations. Notably, the biosynthetic machinery responsible for assembling this characteristic lactone scaffold has been identified in several plant families, including Lauraceae, Cupressaceae, and Linaceae. Among Lauraceae, the aryltetralin lignan (−)-morelensin, isolated from *O. macrophylla*, shares the same 8–8′, 2–7′ biosynthetic origin as podophyllotoxin but differs structurally by possessing only two methoxy substituents on its aromatic rings, in contrast to the three groups in podophyllotoxin.^53,84–86^

Regarding the chemical cores of lignoids, their general structural classification is based on the nature of the intermolecular linkage that joins the two phenylpropanoid (C_6_–C3) units. These linkages may occur through carbon–carbon (C–C) bonds—as observed in lignans and neolignans—or through carbon–oxygen–carbon (C–O–C) bridges, giving rise to oxyneolignans.^19,39,83^ Among the *Ocotea* species reported in phytochemical studies in the literature, ∼11.5% (n = 21) produce lignans, while ∼87% (n = 158) have at least one neolignan in their metabolome. Oxyneolignans are less frequently encountered, occurring in only ∼1.6% (n = 3) of studied species, including *O. cymosa* and *O. costulatum*.^87^ This profile suggests an evolutionary shift or specialization within *Ocotea* toward neolignan-dominated metabolism, possibly a result of genomic expansion of biosynthetic genes regulating phenylpropanoid coupling. *Ocotea* species express a broad set of oxidative enzymes (e.g., peroxidases, laccases) and dirigent proteins (DIRs) that guide the stereoselective radical coupling of monolignols into nonclassical C_6_–C_3_ dimers of the neolignan scaffolds. Unlike strict 8–8′ linkages in lignans, these enzymes favor alternative coupling positions (8–1′, 8–3′, 7–3′, etc.), enabling formation of complex neolignan scaffolds such as benzofurans and bicyclic octane cores.

Regarding the lignan scaffolds, the 7–7′ epoxylignans (furan lignan, **C2**, Figure 6) and 9–9′ diepoxylignans (furofuran lignan, **C3**) represent the most abundant scaffolds in the *Ocotea* genus, together accounting for approximately 76% of all lignans reported for *Ocotea* species in the phytochemical literature. The derivatives of the bicyclic octane neolignans are encountered in over 60% of the lignoids *Ocotea* species, such as *O. macrophylla*, *O. catharinensis*, *O. porosa*, *O. cymosa*, and others (Supplementary Table S3).^88–91^ Lignoids in general represent the second most common class of specialized metabolites in the *Ocotea* genus, characterized in 18 out of 115 different *Ocotea* species already chemically studied.^19,88–90,92–96^ Our recent publication, which employed modern chemical annotation for screening several *Ocotea* species not chemically profiled before, increased the number of known *Ocotea* species able to elicit lignoid biosynthesis to 45 (Supplementary Tables S3 and S3.1).^16^

**Figure 6:**
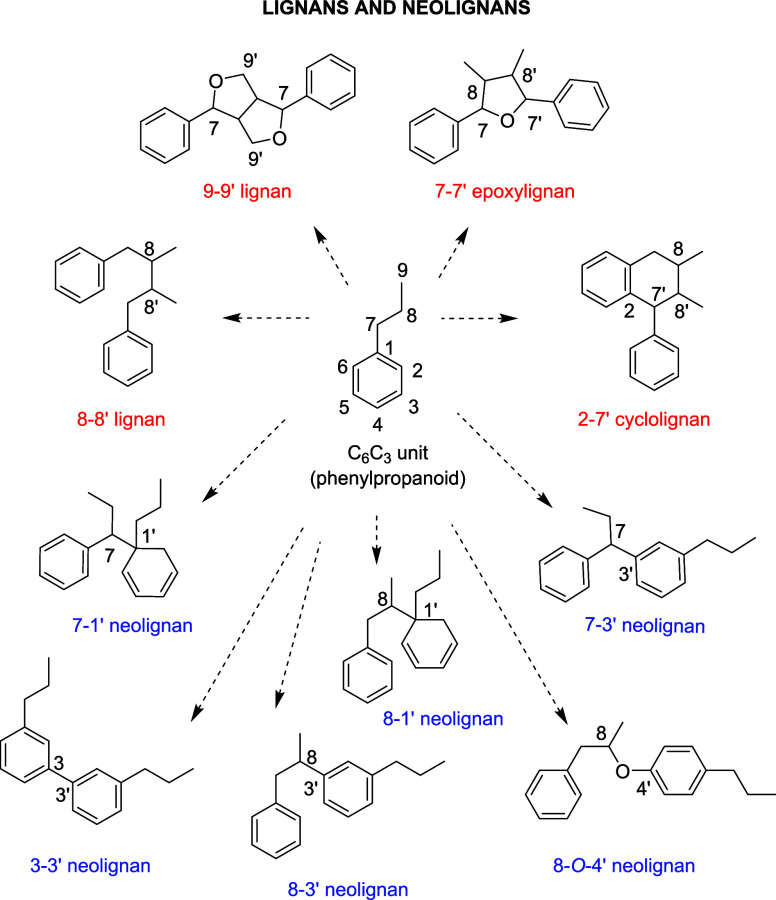
C_6_–C_3_ units of a phenylpropanoid core and examples of the difference between lignans and neolignans based on different potential linkages.

Figure 6 illustrates Haworth’s (1937) definition of a lignan, which remains widely accepted today. According to this definition, lignans arise from the oxidative dimerization of two C_6_–C_3_ units connected at the 8–8′ (β–β′ linked) positions, with possible variations at the 7–7′, 9–9′, and 2–7′ positions. Linkages deviating from this pattern, such as 3–3′, 7–1′, 7–3′, 8–1′, and 8–3′ linkages or ether linkages such as 8-*O*-4′, are classified as neolignans.^39,53,83^

Later, Gottlieb (1974) suggested that the classification can be based on their respective biosynthetic precursors, helping to rationalize the formation of different coupling patterns.^97^ Thus, the basic lignoid core is formed through the oxidative coupling of phenylpropanoids, yet the plant’s metabolism shows a degree of biosynthetic independence, resulting in various congeners. The term “lignans” should be retained for derivatives condensed via the oxidative coupling of cinnamyl alcohol and/or cinnamic acid (8–8′ linked). Neolignans, on the other hand, are derived via condensation and oxidative coupling between propenylphenols and allylphenols, typically via non-β–β′ linkages. More recently, the Lignan Handbook published by Lewis and collaborators (2022) has argued that this distinction should be revised, recommending abandonment of the prefix “neo-” because these compounds arise from the same phenylpropanoid biosynthetic origin and differ mainly in their coupling patterns.^98^ This view echoes earlier considerations by Gottlieb that they are not “different pathways”, but different substrate pools + oxidative coupling. Previously, Gottlieb and Yoshida (1984) had indicated a similar perspective, but at that time, the academic community, particularly organic synthetic chemists, was already employing the neolignoid nomenclature.^99^

In the present review, the traditional lignan and neolignan terminology was retained to preserve consistency with the primary literature and with the organization of the chemical records compiled in *Ocotea*DB, thereby enabling direct comparison and traceability across published reports on *Ocotea* spp. Nevertheless, recent proposals for revising this nomenclature are acknowledged, and the biosynthetic relationships among these lignoids are discussed here in an integrated manner. Accordingly, these classes of compounds are synthesized in plants via the shikimate pathway, utilizing as precursor the *p*-coumaric acid (**10**), which serves as a critical core in the phenylpropanoid pathway and has been isolated from *O. minarum*.^100,101^ From this pivotal intermediate, a tightly regulated sequence of hydroxylation, methylation, reduction, and oxidative coupling reactions gives rise to the diverse lignoid scaffolds discussed herein (Figure 7). Collectively, this metabolic convergence underscores the phenylpropanoid pathway as a central biosynthetic platform from which a wide spectrum of structurally and biologically distinct lignoids and related specialized metabolites emerge.

**Figure 7:**
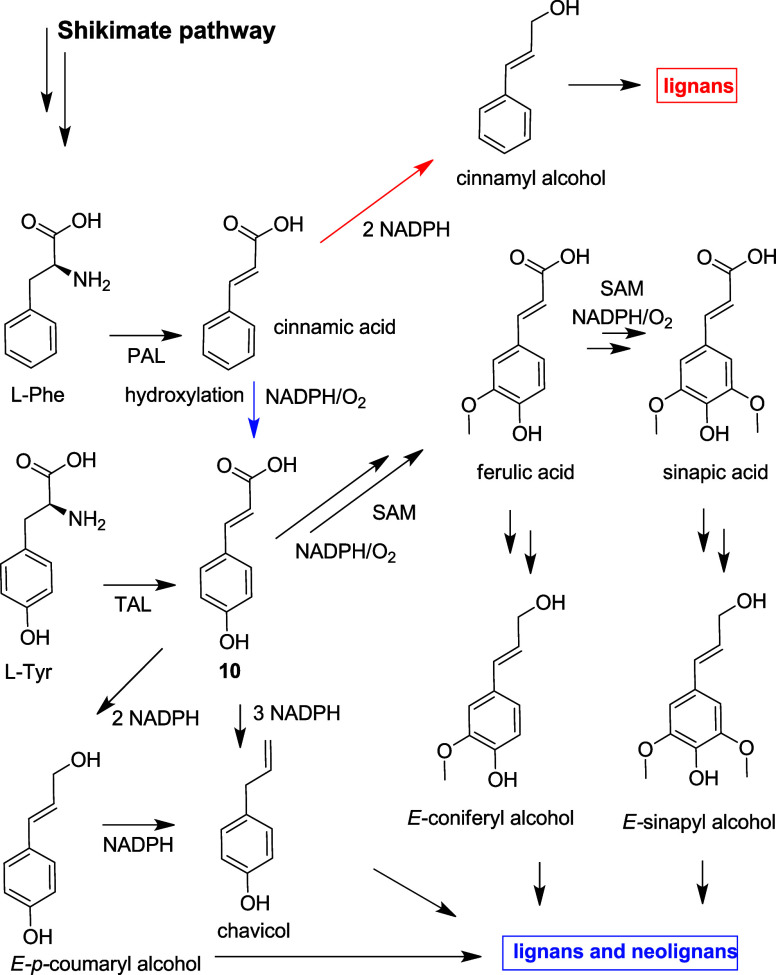
Primary monomers are derived from the shikimate pathway in the biosynthesis of lignans and neolignans. Following the formation of *p*-coumaric acid (**10**), a cascade of biosynthetic reactions is initiated within the phenylpropanoid pathway.

The biosynthesis of lignoids frequently results in enantiomerically pure metabolic products, attributed to the control exerted by stereoselective enzymes during the coupling reactions that form lignans and neolignans. Examples include the oxyneolignan (*S*)-virolongin B (**C7.2**) and (*S*)-ococymosin (**C6.3**), both neolignans isolated from *O. cymosa*,^87^ showcasing the predominant optically active forms, similar to certain alkaloids. However, it is important to mention that these NP can also be isolated as racemic or enantiomeric mixtures.^102–104^ Reactions typically catalyzed by laccase (Lac) or peroxidase (Pex) enzymes, often guided by dirigent DIR-proteins, that control the regioselectivity and enantioselectivity of the β–β′ linkage, lead to the formation of the furofuran lignan (+)-pinoresinol, a 9–9′-diepoxylignan. Subsequently, the furofuran syringaresinol (**11**) and yangambin (**12**) are derived from this through enzymatic hydroxylation and SAM/OMT methylation. Compounds **11** and **12** have been described in *O. duckei*, and **12** also in *O. heterochroma*, respectively (Supplementary Table S3; Figure 8).

**Figure 8:**
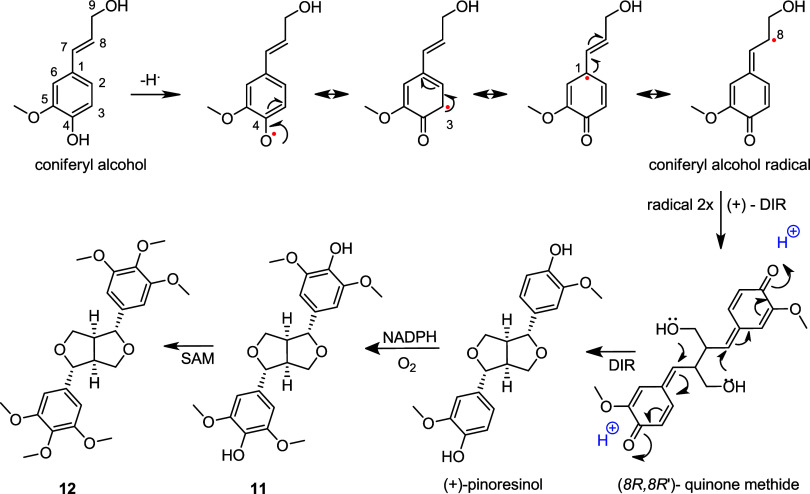
Coniferyl alcohol radicals and DIR enantioselective formation of (+)-pinoresinol and related lignoids syringaresinol (**11**) and yangambin (**12**).

The furan lignans (e.g., 7–7′-epoxylignan cores) are formed by the coupling of propenylphenol monomer units, which are coniferyl alcohol derivatives, such as eugenol (**13**) and isoeugenol (**14**). DIR-guided radical dimerization of *E*-isoeugenol gives the characteristic 7,7′-epoxylignan (+)-verrucosin, which functions as a central biosynthetic intermediate in Lauraceae. Subsequent SAM-dependent *O*-methyltransferase reactions on this verrucosin-type scaffold generate the closely related furan lignans such as (+)-veraguensin (**15**) and feasibly the (+)-methylverrucosin (**16**), as they all share a common 2,5-diaryl-3,4-dimethyltetrahydrofuran core derived from oxidative coupling of eugenol- or isoeugenol-type propenylphenols (Figure 9).

**Figure 9:**
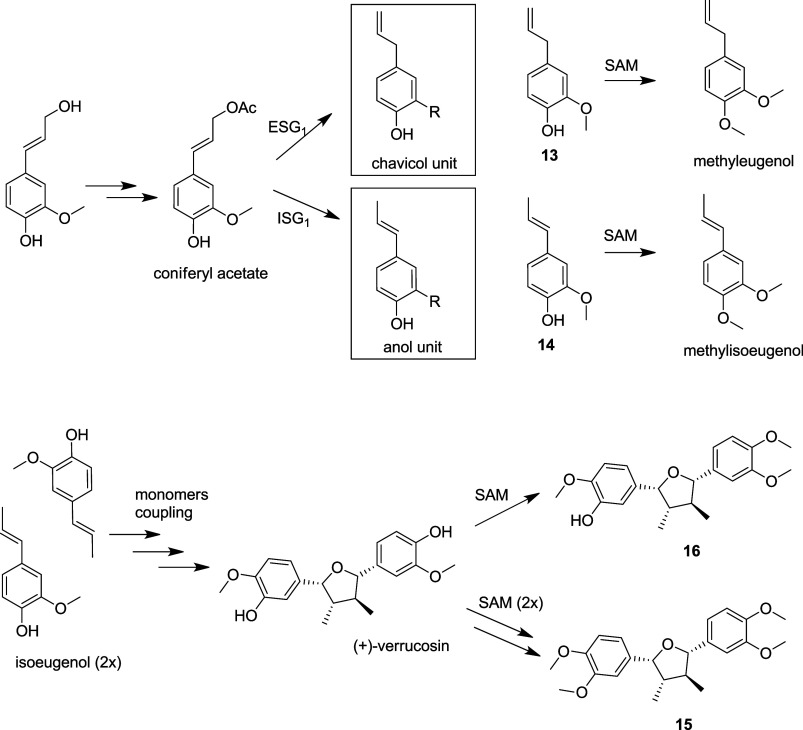
Proposed (+)-verrucosin, (+)-veraguensin (**15**) and (+)-methylverrucosin (**16**) biosynthesis via isoeugenol (**14**) and derivative precursors.

These examples highlight the crucial role of configuration in lignoid biosynthesis, as several lignan subclasses incorporate four or more stereogenic centers formed during oxidative coupling and downstream tailoring steps. Among these structures, the furan lignan cores corresponding to compounds **11** and **12** are among the most frequently reported in the *Ocotea* genus isolated from *O. foetens*, *O. veraguensis*, *O. catharinensis*, and several additional *Ocotea* species (Supplementary Table S3). The conversion of coniferyl acetate in the chavicol and anol units of phenylpropene, such as eugenol and isoeugenol, is catalyzed by NADPH-dependent reductases and eugenol and isoeugenol synthases, respectively (Figure 9).^100,105^

The neolignan scaffolds are abundant in the Lauraceae, particularly in the *Ocotea* genus (Supplementary Table S3). However, even though lignoids display diversity, within the *Ocotea* genus, more than one-third (48.4%, n = 88) are bicyclic neolignoids, making this subclass the most representative lignoid subclass in the *Ocotea* genus. These compounds have been identified in different species, such as *O. aciphylla*, *O. bullata*, *O. catharinensis*, *O. veraguensis*, and *O. porosa*, among others.^88,90,94,95,106,107^ Following closely, the benzofuran subclass accounts for 31.3% (n = 57) of the isolated lignoids, found in species such as *O. catharinensis*, *O. veraguensis*, *O. porosa*, and *O. macrophylla*, among others (Supplementary Table S3). The high prevalence of these two subclasses within *Ocotea* spp. is not merely coincidental. Considering biosynthetic aspects, benzofuran neolignans, particularly those with 8–1′ and 8–3′ linkages, are considered key precursors in the biosynthetic pathway before rearrangement into bicyclic octane neolignoids. Figure 10 illustrates the acid-catalyzed rearrangement of the 8–1′ linkage to form the classical bicyclic neolignoid scaffold. Additionally, the reverse pathway to the benzofuran core is feasible, allowing for further biochemical modifications within the plant biosynthesis.^96,99,108^

**Figure 10:**
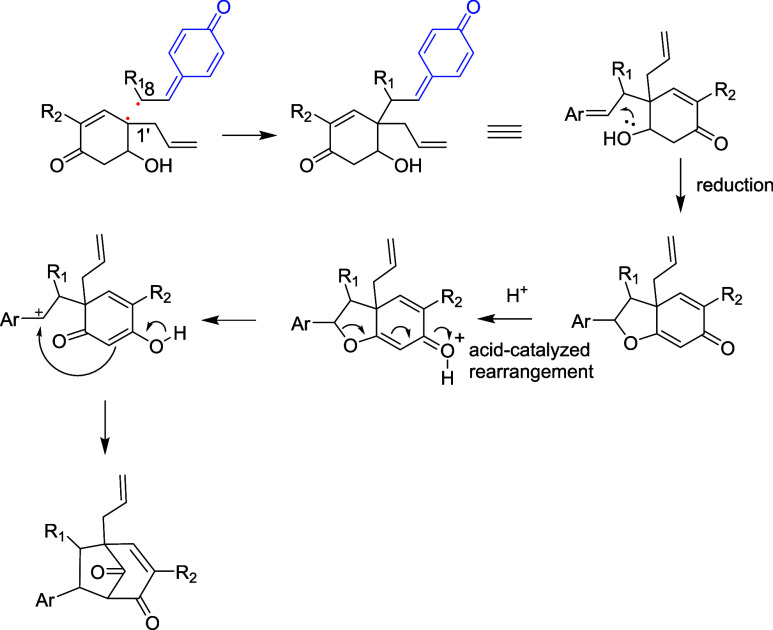
Proposed bicyclic octane neolignoids via 8–1′ linkage radical monomers based on an acid-catalyzed rearrangement mechanism.

### Lignoid Profile

Over the past century, lignoids have been extensively studied in terms of their role as phytochemical biomarkers through various systematic approaches, prominently by Gottlieb and collaborators (from 1970 to 2000).^8,89,90,92–94,96,97,99,108^ Recently, a metabolomics study using only literature data by Antonio et al. (2020) showed a shift in academic interest toward alkaloids in the 21st century, with lignoids receiving less attention. Nevertheless, despite their research limitations regarding the acquisition of data for metabolomics models and the limited available data on lignoids from *Ocotea* spp., their multivariate analysis corroborated that *Ocotea* lignoids are valuable for chemophenetics. In addition, according to their work, Gottlieb’s statement that *Ocotea* species exclusively biosynthesize either alkaloids or lignoids is no longer valid, as modern chemical analytical techniques have indicated that different *Ocotea* species can elicit both alkaloids and lignoids (e.g., in the case of *O. macrophylla*, *O. duckei*, and *O. minarum*).^19^

In this context, the study of metabolomics features through untargeted metabolomics strategies has the potential to enhance plant taxonomic issues in the genus. Species differentiation could be rapidly achieved using metabolic fingerprints obtained from state-of-the-art analytical tools such as UPLC-HR-ESI-MS.^109,110^ Antonio et al. (2020) also demonstrated that comparing lignoid contents is effective in distinguishing between similar *Ocotea* species due to the significant variation in the lignoid profiles. Furofuran lignans, such as **12**, have proven to be effective phylogenetic biomarkers within the genus.^19^ The continued application of untargeted metabolomics may uncover new markers, aiding taxonomic classifications, as these methods are already widely used in drug and phytomedicine quality control.^111–113^

Increasing focus on the chemical data and bioactivity of lignoids over the past decade suggests that these compounds may soon regain broader interest within the academic community. Original articles accessing chemical data and the bioactivity of lignoids have increased over the past 10 years. Notably, pharmacological effects have been reported in approximately 20% of the chemically characterized *Ocotea* species. To date, 182 lignoid chemical structures have been isolated (and curated) from the genus (Supplementary Table S3). Figures 11,12,13,14,15,16 present the lignoid cores found among the different *Ocotea* lignoid producers reported in the literature.

**Figure 11:**
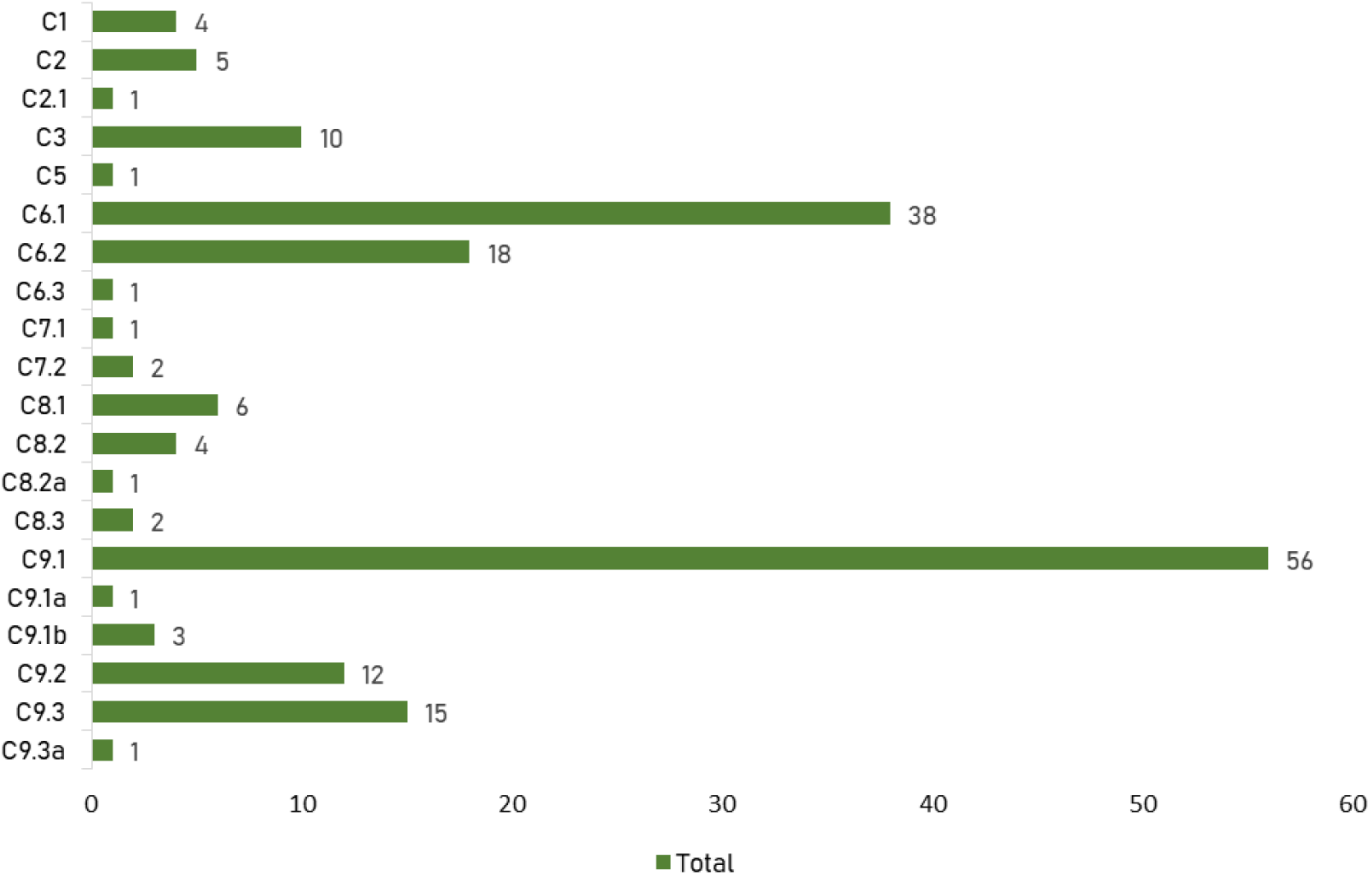
Chemical diversity of lignoids in the *Ocotea* genus, expressed as the number of different chemical structures reported in the literature.

**Figure 12:**
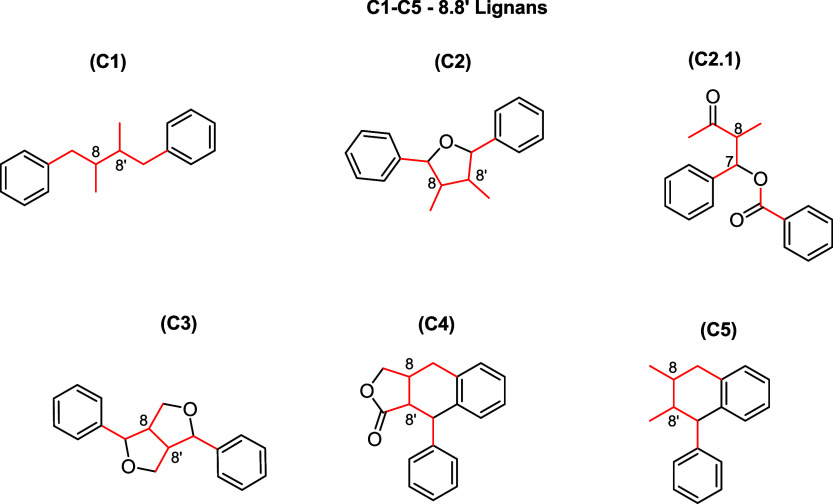
Lignan chemical scaffolds found in *Ocotea* species: (**C1**) 8–8′-lignans, (**C2**) 7–7′-epoxylignans, (**C3**) 9–9′-diepoxylignans, (**C4**) 8.8′,2.7′-aryltetralin lignan lactones, and (**C5**) 2–7′-cyclolignans. The molecular diversity of lignans in the genus *Ocotea* is expressed by the number of different chemical structures for each subclass of lignan scaffolds.

**Figure 13:**
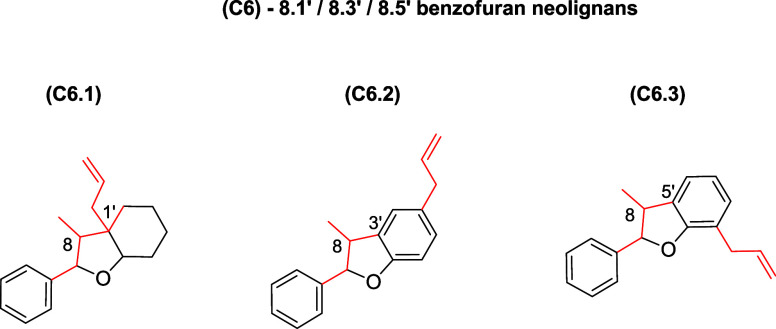
Benzofuran neolignan chemical scaffolds found in *Ocotea* species: (**C6.1**) 8.1′-, (**C6.2**) 8.3′- and (**C6.3**) 8.5′-benzofuran neolignans.

**Figure 14:**
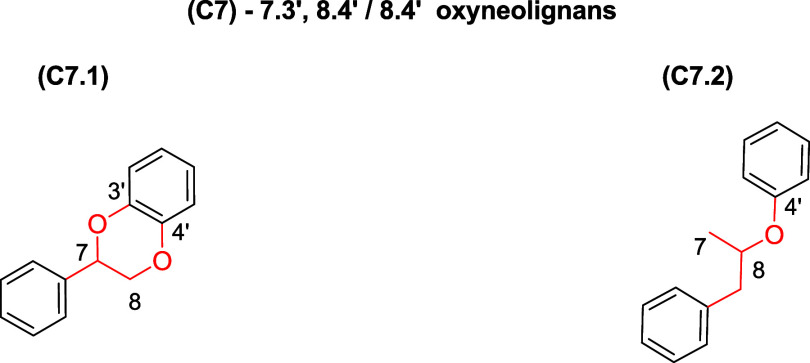
Oxyneolignan chemical cores found in *Ocotea* species: (**C7.1**) dioxyneolignan and (**C7.2**) oxyneolignan.

**Figure 15:**
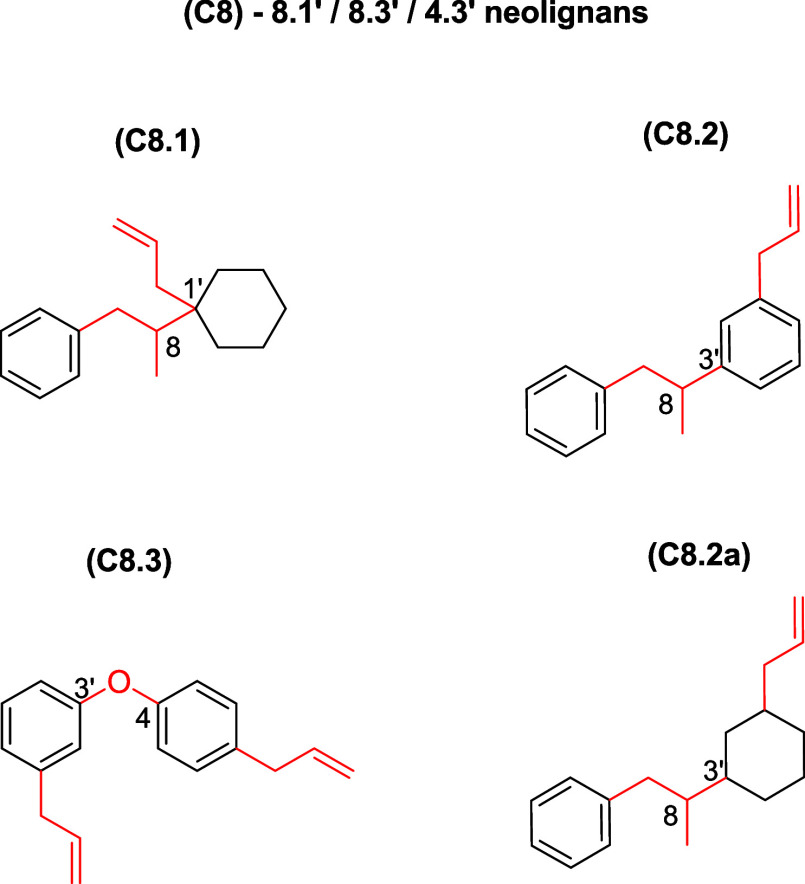
Lignoid chemical scaffolds found in *Ocotea* species: (**C8.1**) 8.1′-neolignan, (**C8.2** and **C8.2a**) 8.3′-neolignan and (**C8.3**) 4.3′-didymochlaenone neolignans.

**Figure 16:**
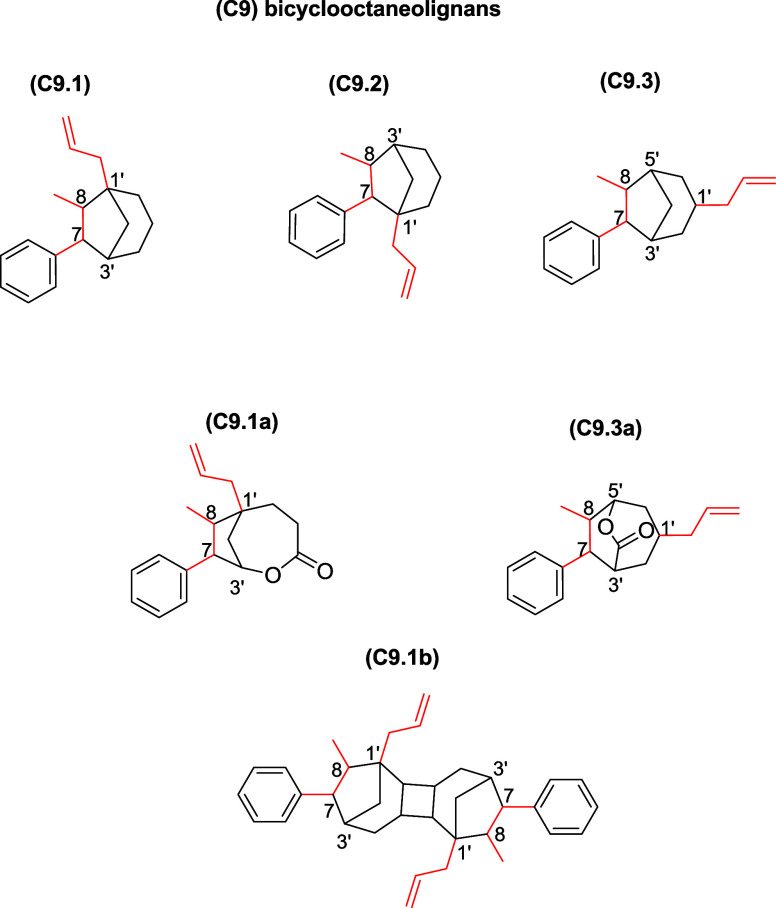
Bicyclic neolignan chemical cores found in *Ocotea* species: (**C9.1**) and (**C9.1a**) 7.3′, 8.1′-connected and (**C9.1b**) 7.3′, 8.1′-dimer. (**C9.2**) 7.1′, 8.3′-connected. (**C9.3**) 7.3′, 8.5′-connected bicyclo[3.2.1]­octanoid and (**C9.3a**) 7.3′, 8.5′ oxabicyclo[3.2.2]­nonane-type neolignans.

*Ocotea* species-produced lignoids have exhibited a high potential for treating or preventing neglected tropical diseases (NTDs) such as dengue fever and leishmaniasis, which affect millions of people in vulnerable situations worldwide.^114^ Accordingly, potent larvicidal and antiprotozoal effects have been reported for different lignoids isolated from plants of the *Ocotea* genus. For example, neolignans isolated from *O. cymosa* have shown a high larvicidal potential, halting the *Aedes aegypti* life cycle at the larval stage with 100% mortality.^87,115^ The lignan yangambin (**12**, **C3**) and the neolignan licarin A (**C6.2**) from *O. macrophylla* have exhibited low IC_50_ values for amastigote and promastigote forms of different *Leishmania* (L.) subspecies. In addition, **12** and burchelin (**C9.2**) have been reported in the literature as highly cytotoxic agents against trypomastigote forms of *T. cruzi*.^116–121^

Moreover, lignoids are recognized as compounds with notable pharmacological properties, including pronounced antitumor, antiviral, and anti-inflammatory effects.^4,39,86,122,123^ For instance, reports have indicated the activity of furofuran lignans (**C3**), aryltetralin lactones (**C4**), and cyclolignans (**C5**) against a broad spectrum of cancer cell lines, including human lung adenocarcinoma (A-549), human colon adenocarcinoma (HT-29), human breast adenocarcinoma (MCF-7), and murine lymphocytic leukemia (P-388).^84^ Regarding antiviral activities, different classes of lignoids have been proven effective against hepatitis B virus (HBV), human cytomegalovirus (HCMV), and human immunodeficiency virus (HIV), severe acute respiratory syndrome-related coronavirus (SARS-CoV), and Zika virus (ZIKV).^86,122^ Additionally, diastereomeric lignans isolated from *O. macrophylla* have exhibited PAF antagonism and the potent dual inhibition of COX-2/5-LOX pathways.^106^ A study by our research group has confirmed the potent *in vivo* anti-inflammatory activity of the bicyclic octane neolignans (**C9**) via the dual inhibition of prostaglandin E2 (PGE2) production and neutrophil recruitment. However, they were isolated from *Aniba firmula*, which also belongs to the Lauraceae family.^91,123,124^ The derivatives of the bicyclic octane neolignans are encountered in over half of the lignoid-producer *Ocotea* species, such as *O. macrophylla*, *O. catharinensis*, *O. porosa*, *O. cymosa*, and others (Supplementary Table S3).^88–90,106^ Furthermore, the bicyclic neolignoid sibyllenone (**C9.2**), isolated from *O. bullata*, has shown promising anti-inflammatory activity via the inhibition of 5-LOX enzymes.^107^

Finally, Teppono et al. (2016) have reported advances in lignoid pattern biosynthesis, detailing a classification into 10 lignan core subtypes and 15 neolignan subtypes.^83^ Our review highlights that the *Ocotea* genus encompasses 5 of these 10 lignan subtypes: 8–8′-lignans (**C1**), 7–7′-epoxylignans (**C2**), 7–8-secolignans (**C2.1**), 9–9′-diepoxylignans (**C3**), 8.8′, 2.7′-aryltetralin lignan lactones (**C4**), and 2–7′-cyclolignans (**C5**). Additionally, the genus hosts four different neolignan core subtypes, including 8.1′-, 8.3′- and 8.5′-benzofuran neolignans (**C6**), 7.3′-, 8.4′-/8.4′-oxyneolignans (**C7**), 8–1′-/8–3′-/4–3′-neolignans (**C8**), and bicyclic octane neolignans (**C9**). Thus, alongside its alkaloids, *Ocotea* is also a remarkably diverse source of lignans and neolignans, offering significant variations in scaffold structures (Figures 11–16).

### *Ocotea* spp. Flavonoids

Flavonoids are specialized metabolites characterized by two benzene rings linked by a pyran moiety, which can undergo a wide variety of chemical modifications. These include substitutions with hydroxyl, methoxy, or glycosyl groups, which significantly affect their physical and chemical properties and their biological activities. Flavonoids also play a crucial role in plant ecology, acting as defense agents against several environmental and biotic stresses, such as ultraviolet radiation, pests, and diseases, as well as beneficial effects on human health in terms of their antioxidant, anti-inflammatory, anticancer, and antiviral properties.^125–129^ In addition, these compounds represent a diverse group of naturally occurring polyphenolic metabolites prevalent in the plant kingdom, including Lauraceae and, consequently, the *Ocotea* genus.^4,19,130,131^

In this review, flavonoids were identified as the third most commonly reported metabolite class in the *Ocotea* genus, with 61 different described metabolites distributed in 20 species. However, in our recent publication using modern annotation analytical techniques, this number was significantly increased to 60 *Ocotea* species (Supplementary Tables S1.1–1.2, S4 and S4.1).^16^

The analysis of flavonoid chemical cores can aid in taxonomic classifications and fill potential phylogenetic gaps. By identifying specific flavonoid subclasses, investigations in the fields of chemophenetics, metabolomics, and transcriptomics can be implemented to elucidate the relationships between different *Ocotea* spp. and other genera.^19^ Biosynthetically, the carbon chain is named as the C_6_–C_3_–C_6_ unit coming from both phenylpropanoid (C_6_–C_3_) and acetate (3 × C_2_) metabolism (Figure 17).^53,125^ Such molecules can exhibit chirality and interesting bioactivity, and the flavanone core serves as the central precursor for downstream subclasses such as flavones and flavonols. The latter being the most abundant in *Ocotea*, particularly those based on kaempferol (**17**) and quercetin (**18**), with myricetin occurring less frequently, and the former apigenin (**19**) and luteolin (**20**).^19,53,125,130,132^

**Figure 17:**
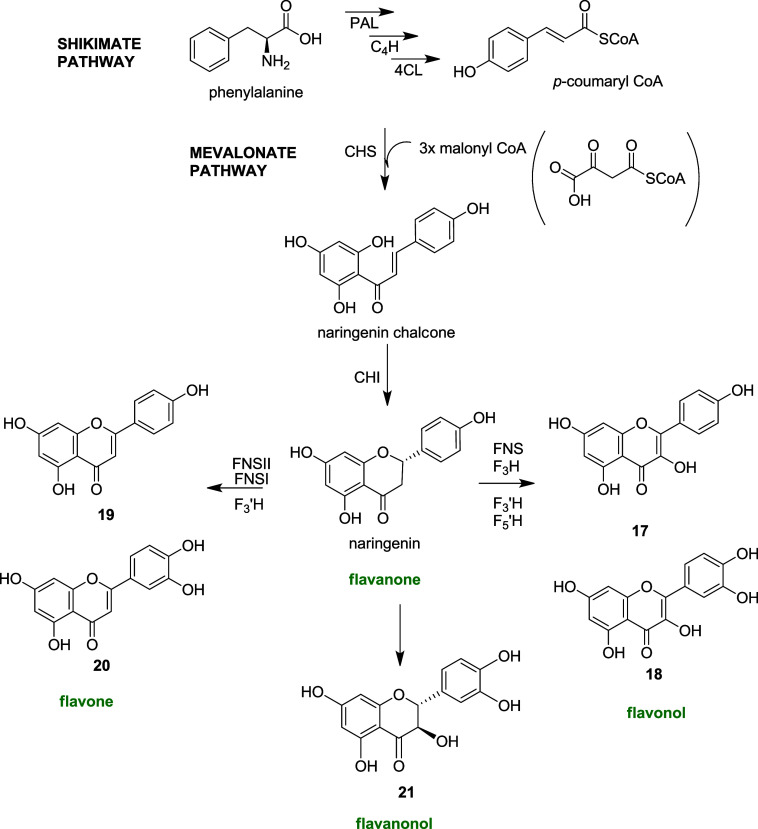
General biosynthesis of *Ocotea* flavonoids, including flavanone (naringenin), flavonol (**17** and **18**), flavone (**19** and **20**), and flavanonol (**21**).

Beyond the initial transformations summarized in Figure 17, extensive enzymatic diversification can expand the structural complexity of flavonoids.^53,125^ For example, flavonolignans represent a hybrid class of NP biosynthesized through the oxidative coupling of flavonoids such as taxifolin (**21,** 5,7,3′,4′-flavanonol) with a phenylpropanoid, typically coniferyl alcohol precursor. Recently, a flavonolignan, mururin A was isolated for the first time within the *Ocotea* genus, specifically from *O. diospyrifolia*.^57^

### Flavonoid Profile

In the *Ocotea* genus, flavonoids are predominantly derivatives of the flavonols **17** and **18**.^19^ Despite growing research interest, the knowledge on *Ocotea* flavonoid profiles remains limited.^130,133^ To date 61 distinct flavonoids have been reported, of which ∼ 18% are nonsubstituted flavonoid cores (n = 11), while the majority ∼67% (n = 41), are glycosylated derivatives; about ∼8% (n = 5) are of lactonized cores, and another ∼8% (n = 5) are catechin or flavonol dimers. Among the glycosylated ones, ∼59% of the total (n = 36) are *O*-glycosylated, and only ∼8% (n = 5) are *C*-glycosylated derivatives. *O*-alkylated flavonoids have so far been reported in only four species, including *O. canaliculata*, *O. caudata*, *O. guianensis* and *O. corymbosa*, whereas *C*-glycosylated derivatives have been found in *O. porosa*, *O. aciphylla*, *O. foetens*, *O. nutans*, and *O. odorifera* (Tables S4 and S4.1).

A recent publication^130^ regarding flavonoid profile using UPLC-HRMS indicated the presence of an apigenin flavone chemical core in six other *Ocotea* species, including *O. diospyrifolia*, *O. guianensis*, *O. lancifolia*, *O. notata*, *O. odorifera*, and *O. porosa*. The apigenin derivatives were found mainly in *C*-glycoside form, with *O. porosa* exhibiting the highest flavone:flavonol ratio. Taken together, the predominance of *O*-glycosylated flavonoids in the genus, the low occurrence of *O-*alkylated flavonoids and the annotation of *C*-glycosylated apigenin derivatives in different species suggest a basal or intermediate phylogenetic position for *Ocotea* within the Lauraceae, as previously pointed out by Antonio et al. (2020).^19^ This metabolic signature can reflect a transitional evolutionary stage, where simpler glycosylation patterns precede the complex methylation seen in other Lauraceae genera. This is supported by the high occurrence of *O*-alkylated flavonoids in evolutionarily advanced plant groups. However, as phylogenetic studies increase in accuracy and precision, with a larger set of evaluated species, additional studies using DNA barcoding together with morphological data could provide further evidence to support or refute this hypothesis.^24^

Flavonoids are not only crucial from an evolutionary and phylogenetic perspective but are also noted for their diverse bioactivities. The most common flavonoid core found in *Ocotea* species is the 3-*O*-glycoside flavonol (**C3**)—isoquercitrin, which constitutes a representative example, which has already been isolated from *O. notata*, *O. elegans*, *O. corymbosa*, and *O. caudata*. Isoquercitrin has been shown to possess antibacterial activity against *Escherichia coli* cells, inducing apoptosis-like death and damaging membrane dynamics by inducing oxidative stress.^134^ This underscores the potential bioactivity of *Ocotea* flavonoids, which warrants further exploration of their biological properties. Flavonoids have also been increasingly recognized for their antioxidant and pro-oxidant properties, as well as for relevant antiviral and antiprotozoal effects.^78,135^ Flavonols can be used to seek more effective treatments for NTDs, such as leishmaniasis and trypanosomiasis.^136^ In addition, *Ocotea* species such as *O. minarum* and *O. odorifera* have been recognized as potent natural antioxidants.^101,137^ Studies have reported antiviral effects against the herpes virus (HSV) and antibacterial effects for flavonoids of the *O. notata* leaves extract.^4,138^ Recently, bacteriostatic effects were evidenced for *O. minarum* flavonoid fractions against the *Salmonella*, *Pseudomonas*, and *Proteus* genera.^101^ Furthermore, biflavonoids (**C10**) such as those found in *O. odorifera* and *O. canaliculata*, might be able to reduce the viability of tumor cell lines, as previously reported.^139^

Besides, it is important to highlight that flavonoids, as other metabolites containing catechol units, are on the list of compounds with potential assay interference (PAINS). This is because they can provide promiscuous behaviors on screening assays since catechols can chelate metals as well as being reactive in the oxidized form to nucleophiles present in the side chains of proteins, interfering with assays or providing nonspecific activities. Thus, interpretation of the biological activities of flavonoids and their derivatives requires scrutinous analysis based on investigation of specific mechanisms of action.^140^

A broad range of specific flavonoid glycoside derivatives was isolated from several *Ocotea* spp., such as *O. vellosiana*, *O. odorifera*, *O. porosa*, *O. notata*, *O. lancifolia*, and others.^4,19^ Figures 18 and 19 present all the different flavonoid cores encountered in the genus, including flavones (**C1**), flavonols (3-hydroxyflavone or flavonol, **C2**), 3-*O*-glycoside flavonols (**C3**), 7-*O*-glycoside flavone-flavonols (**C4**), and flavanonols (**C5**). In addition, the 3-*O*-glycoside flavanonols (**C6**), flavan-3-ols (**C7.1**), phenylpropanoid-substituted flavan-3-ols (**C7.2**), 7-*O*-glycoside flavanones (**C8**), 8-glycoside flavones (**C9.1**), 6-glycoside flavones (**C9.2**), flavanonol dimers or biflavonoids (**C10**), tetrahydroxychalcones (**C11**), and flavonolignans (**C12**) are included.

**Figure 18:**
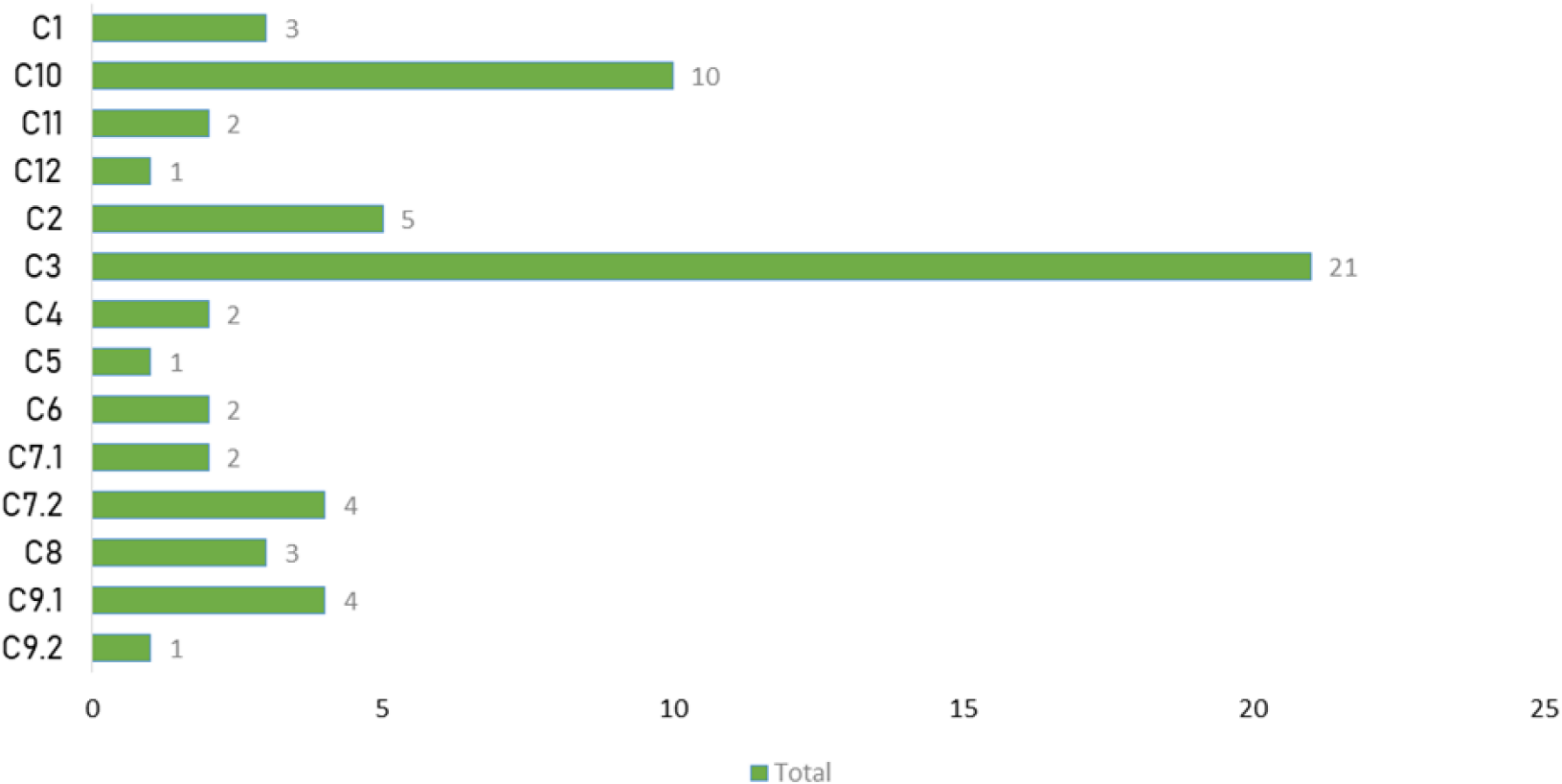
Chemical diversity of flavonoids in the *Ocotea* genus is expressed as the number of different chemical structures reported in the literature.

**Figure 19:**
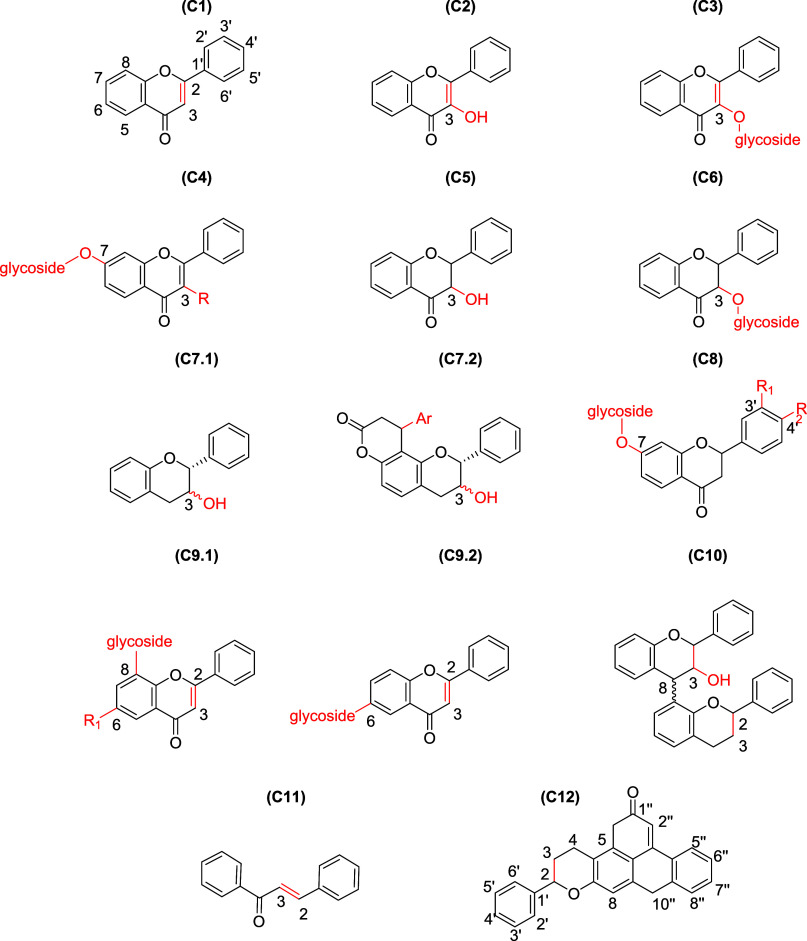
Flavonoid cores found in *Ocotea* species: (**C1**) flavone, (**C2**) flavonol, (**C3**) 3-*O*-glycoside flavonol, (**C4**) 7-*O*-glycoside flavonol, (**C5**) flavanonol, (**C6**) 3-*O*-glycoside flavanonol, (**C7.1**) flavan-3-ol, (**C7.2**) phenylpropanoid-substituted flavan-3-ol, (**C8**) 7-*O*-glycoside flavanone, (**C9.1**) 8-glycoside flavone, (**C9.2**) 6-glycoside flavone, (**C10**) flavanonol dimer or biflavonoid, (C11*) tetrahydroxychalcone, and (**C12** ^#^) flavonolignan. *Flavonoid precursor; ^#^flavonoid mixed biosynthetic class.

### *Ocotea* spp. Terpenoids

Terpenoids represent the largest classes of chemicals found in the essential oils of *Ocotea* spp., followed by phenolic compounds, such as phenylpropanoid and phenylpropene derivatives, along with aldehydes, alcohols, and esters. Indeed, volatile terpenoids are the predominant constituents in the essential oils of a vast array of plants and flowers globally.^141–143^ These compounds are biosynthesized via two pathways in superior plants: the mevalonic acid (MVA) and 2-*C*-methyl-D-erythritol-4-phosphate (MEP) pathways.^53^ The *Ocotea* species produce majoritarian sesquiterpenes via MVA and monoterpenes via MEP, indicating by our research that both pathways are highly evolved and active within the *Ocotea* metabolome (Supplementary Tables S5, S6, and S5.1–S6.1).

The isopentenyl diphosphate (IPP) and dimethylallyl diphosphate (DMAPP) are the primary building blocks for terpenoid formation, and assemble in a head-to-tail fashion catalyzed by geranyl pyrophosphate synthase (GPP) to form the chemical core of most monoterpenoids (C_10_), including alyphatic such as geraniol (**22**), citronellol (**23**), and linalool (**24**), and cyclic such as camphor (**25**), borneol (**26**), limonene (**27**), α-terpineol (**28**), and 1,8-cineole (**29**), which are all widespread in the *Ocotea* genus (Figure 20).^13,17,41,57,144–147^

**Figure 20:**
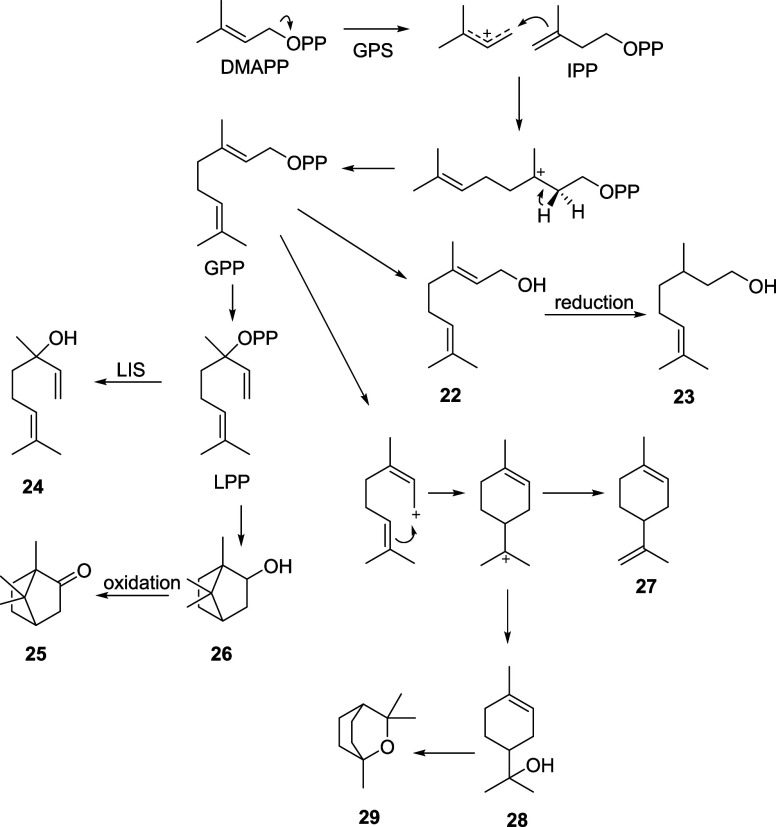
Biosynthesis of geranyl pyrophosphate synthase (GPP), common precursor of monoterpenes including geraniol (**22**), citronellol (**23**), linalool (**24**), camphor (**25**), borneol (**26**), limonene (**27**), α-terpineol (**28**) and 1,8-cineole (eucalyptol) (**29**).

The MVA pathway predominantly leads to the biosynthesis of sesquiterpenes (C_15_), a major class of terpenes formed by the union of three isoprene units. These units originate from the farnesyl diphosphate (FPP) precursor, which is synthesized by the condensation of an IPP unit with GPP.^53^ The addition of the nucleophilic IPP unit to a GPP-derived allylic cation culminates in the formation of the C_15_ farnesyl scaffold. The chain length can be increased by further modifications through oxidative cyclization reactions and rearrangements, giving rise to the largest chemical diversification among terpenoids. Thus, besides the significant number of linear structures, a vast range of sesquiterpene motifs, such as mono-, bi-, and tricyclic structures, can be biosynthesized.^53,148^ The sesquiterpenes (+)-germacrenes A (**30**) and B (**31**) are sesquiterpenes frequently encountered in different *Ocotea* species essential oils (Figure 21).^19,146^

**Figure 21:**
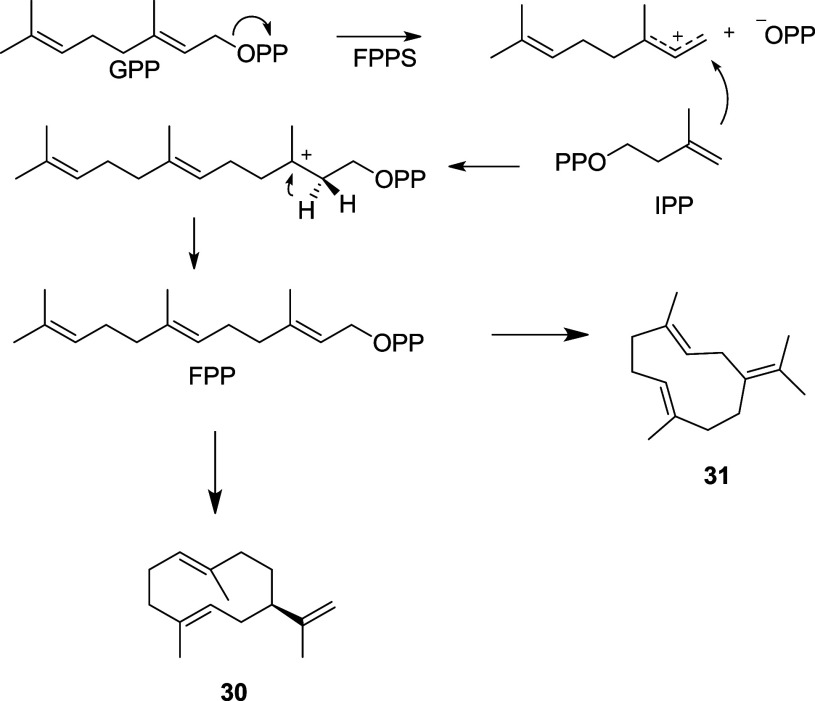
Biosynthesis of FPP, the chemical core for C_15_ derivatives, and sesquiterpenes (+)-germacrene A (**30**) and (+)-germacrene B (**31**).

Diterpenes (C_20_) arise from the head-to-tail condensation of four isoprene units and are biosynthesized from the precursor geranylgeranyl diphosphate (GGPP), which is formed by the sequential addition of one IPP unit to FPP. Additional oxidative modifications catalyzed mainly by terpene synthases and cytochrome P450 enzymes, generate a broad diversity of diterpene skeletons.^53^ However, in *Ocotea*, diterpenes are comparatively uncommon metabolites and have been reported only in a few species, such as *O. floribunda*, *O. neesiana*, *O. nutans*, and *O. bicolor* (Supplementary Tables S5, S6, and S5.1–S6.1). Owing to this limited occurrence, diterpenes are unlikely to represent primary chemotaxonomic markers for the *Ocotea* species. Instead, they may still provide specific chemical profiles useful for the differentiation of species within the genus.

### Terpenoid Profile: Monoterpenoids

*Ocotea* species are known for producing a diverse array of oxygenated and nonoxygenated, linear and cyclic monoterpenes. These compounds are distinguished by their varied chemical structures and unique biological activities, demonstrating a range of pharmacological properties, such as antimicrobial, antifungal, antitumor, and anti-inflammatory effects. Studies have suggested that the therapeutic potential of monoterpenes is due to their ability to interact with various biological targets, including enzymes, receptors, and ion channels. Such interactions can significantly affect cellular processes by modulating signaling pathways and influencing inflammation, apoptosis, and cell growth.^9,145,149–153^

Most phytochemical studies on *Ocotea* report several monoterpenes without enantiomeric assignment, as no attempt to assign the absolute configuration of monoterpenoids has been made in the majority of essential oils chemical investigations reported. Among these, compounds **24**, **26–29**, β-myrcene (**32**), 4-terpineol (**33**), *cis*-β-ocimene (**34**), *trans*-ocimene (**35**), α-pinene (**36**), β-pinene (**37**), and camphene (**38**) are prevalent across various *Ocotea* species (Figure 22). Specifically, monoterpene **32** is not only a major constituent in several plants but is also abundantly found in 22 *Ocotea* species (Tables S6 and S6.1). This compound is a highly important raw material for the production of flavors, fragrances, cosmetics, and also plays an important role in the pharmaceutical field, given its significant biological properties (e.g., anxiolytic, antioxidant, antiaging, anti-inflammatory, and analgesic).^154^ Similarly, **29** (also known as eucalyptol) has been found in 21 *Ocotea* species and is renowned for its anti-inflammatory effects, primarily through the inhibition of interleukins (IL) and interference with tumor necrosis factor alpha (TNF-α) production. It also exhibits antinociceptive, cardiovascular, and vasorelaxant properties.^153,155^

**Figure 22:**
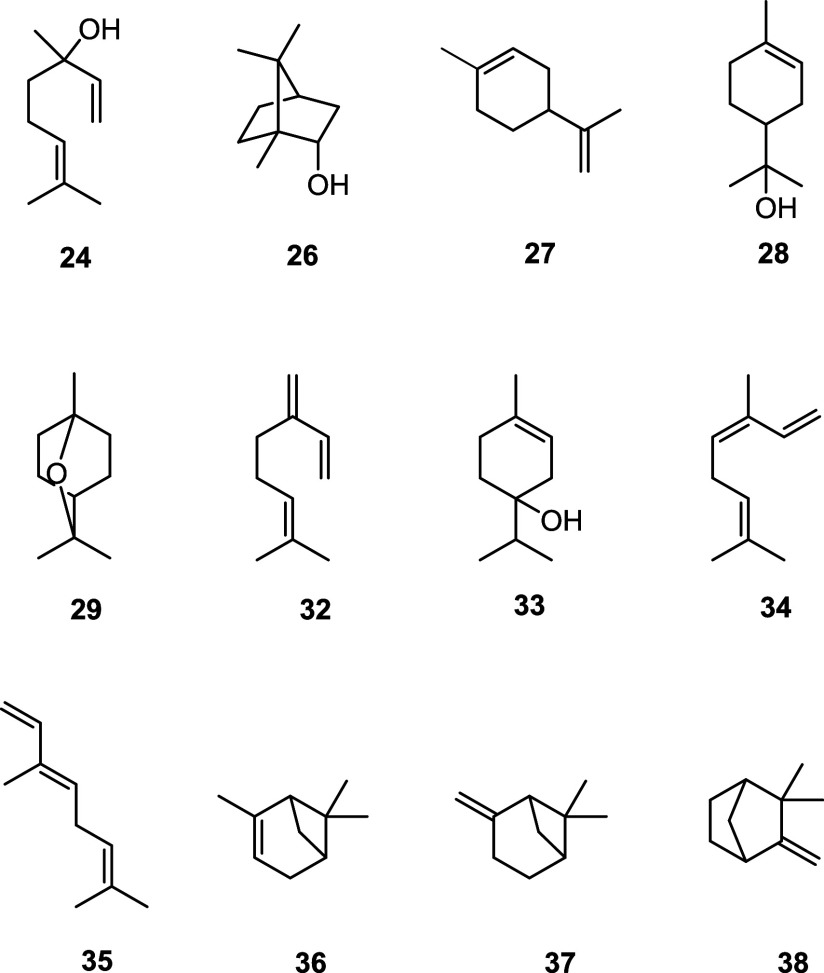
Widespread monoterpenes found in the *Ocotea* genus, including linalool (**24**), borneol (**26**), limonene (**27**), α-terpineol (**28**), 1,8-cineole (**29**), β-myrcene (**32**), 4-terpineol (**33**), *cis*-β-ocimene (**34**), *trans*-ocimene (**35**), α-pinene (**36**), β-pinene (**37**), and camphene (**38**).

Moreover, **29** and **33**, which are commonly found in the tea tree (*Melaleuca alternifolia*), have been identified in 15 *Ocotea* species. These compounds act as anti-inflammatory agents by inhibiting the production of LPS-induced mediators such as IL-1β, IL-6, and IL-10.^156^ **29** is also utilized industrially as an ingredient in aromatic scents, perfumes, and cosmetics, given its lilac-like odor. Additionally, it exhibits a wide range of biological properties, including antioxidant, anticonvulsant, antiulcer, antihypertensive, antinociceptive, and insecticidal effects.^157^ Figure 22 depicts the most commonly identified monoterpenes in the *Ocotea* genus.

Monoterpene linalool (**24**) has been found in 13 *Ocotea* species and is recognized as a biologically active compound of commercial interest due to its beneficial aromatic properties. This compound has demonstrated repellent properties against various crop-destroying insects and potential antioxidant, anti-inflammatory, anticancer, anxiolytic, analgesic, and sedative effects.^158,159^ Moreover, monoterpene borneol (**26**) is a common component in plant essential oils, which is present in numerous medicinal plants, such as *Valeriana officinalis*, *Matricaria chamomilla*, and *Lavandula officinalis*; further, it is also present in 12 *Ocotea* species. In addition, **26** can be used in fragrances and cosmetics and exhibits potential for medicinal applications, pending reports of its biological activities, including its antimicrobial, anti-inflammatory, and antiviral effects.^151,160,161^

Terpenoids from several *Ocotea* species have shown promising antitumor properties against a variety of cancer cell lines, including breast adenocarcinoma epithelial cells (MCF-7 and MDA-MB-231), cerebral glioblastoma (A-172, U-87MG), cerebral astrocytoma (CCF-STTG1), and hepatocellular carcinoma (Hep-G2) cells.^152,162–164^ Moreover, monoterpene α-terpineol (**28**), found in species such as *O. quixos*, *O. bofo*, and *O. opifera* among others, exhibit potent cytotoxic and antitumor effects, mediated through various mechanisms, including the induction of apoptosis, the inhibition of cell migration, DNA fragmentation, and cell cycle arrest.^157^

### Terpenoid Profile: Sesquiterpenoids

Sesquiterpenoids constitute the predominant terpenoid subclasses in the *Ocotea* genus and are major components of the essential oils extracted from various *Ocotea* species. In addition, several terpenoids can also be isolated from different parts of the *Ocotea* species, including barks, leaves, and flowers.^4,29,146,165^ Thus, the most prevalent compounds are presented in Figure 23 and Supplementary Tables S5–6 and S5.1–61, including spathulenol (**39**), α-cadinol (**40**), elemol (**41**), bicyclogermacrene (**42**), caryophyllene oxide (**43**), globulol (**44**), α-humulene (**45**), β-caryophyllene (**46**), 1-*epi*-cubenol (**47**), viridiflorol (**48**), *trans*-nerolidol (**49**), and α-cadinene (**50**).

**Figure 23:**
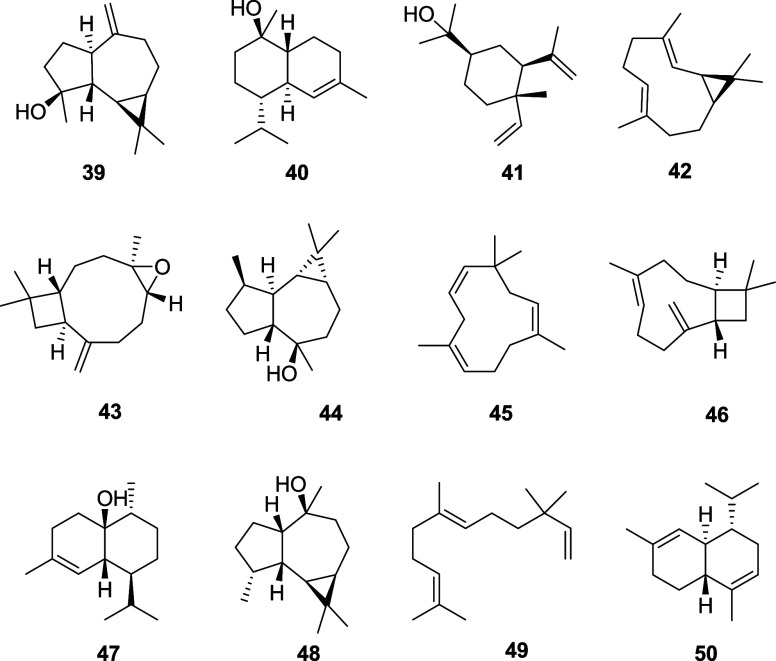
Widespread sesquiterpenes found in the *Ocotea* genus, evidencing spathulenol (**39**), α-cadinol (**40**), elemol (**41**), bicyclogermacrene (**42**), caryophyllene oxide (**43**), globulol (**44**), α-humulene (**45**), β-caryophyllene (**46**), 1-*epi*-cubenol (**47**), viridiflorol (**48**), *trans*-nerolidol (**49**), and α-cadinene (**50**).

Spathulenol (**39**) was identified in 25 *Ocotea* species (e.g., *O. diospyrifolia*, *O. lancifolia*, and *O. nutans*). There are several aromatic medicinal plants in which **39** is recognized as a major volatile constituent, such as *Psidium guineense*, which is also a plant with ethnopharmacological significance and popularly used for treating a range of inflammatory diseases, as well as *O. odorifera*.^56^ In addition to its anti-inflammatory properties, **39** has also exhibited antioxidant, antiproliferative, immunomodulatory, and antibacterial effects.^166–168^

Likewise, α-cadinol (**40**) has been found in 24 *Ocotea* species, alongside other less concentrated isomers. Furthermore, the sesquiterpene **40** and its isomers can be encountered in the essential oils of other plant genera and are recognized for their antioxidant, cytotoxic, and antimicrobial activities.^145,169^ For example, the sesquiterpene **40** and the diterpene 16-methylesteritol A displayed antibacterial potential against *Xanthomonas* with minimum inhibitory concentration (MIC) and minimum bactericidal concentration (MBC) of 50 and 250 μg mL^‑1^, respectively.^170^ Sesquiterpenes elemol (**41**), caryophyllene oxide (**43**), globulol (**44**), β-caryophyllene (**46**), and *trans*-nerolidol (**49**) have been identified in different isomeric forms and belong to the chemical composition of several *Ocotea* species (Supplementary Tables S6 and S6.1). Sesquiterpene viridiflorol (**48**) has been found in 11 *Ocotea* plant species and is noted for its anti-inflammatory action against leukocyte migration and antioxidant and antibacterial activities.^171^

#### *Ocotea* spp. Essential Oils

Essential oils are complex mixtures of volatile organic compounds primarily biosynthesized in specialized plant cells.^14,149,172^ Lauraceae species are highly valued for the essential oils produced by their plants, including those from *Ocotea*.^40^ Despite numerous studies, only 48 of 115 chemically characterized species (∼42.5%) have been examined in terms of their essential oil composition. To date, GC-MS analysis has allowed the identification of 491 different compounds as constituents of *Ocotea* essential oils, including a range of different monoterpenoids (Supplementary Tables S5–6 and S5.1–6.1). These compounds are present in conventional medicine and aromatherapy and are prevalent in cosmetics, dentistry, agriculture, food flavorings, cleaning products, and solvents, making them one of the most successful commodities in industry,^76,141,149,159,173^ exhibiting biological potentials such as antimicrobial, antioxidant, anti-inflammatory, and cytotoxic effects.^146,163,171,172,174–176^

Phytochemically, terpenoids represent the most abundant components in *Ocotea* spp. essential oils, with sesquiterpenoids (64.6%) being the dominant subclass, followed by monoterpenoids (28.5%). The qualitative and quantitative variations in these components can be influenced by factors such as genetic specificity, developmental stages, environmental stress, and adaptations to local climatic and soil conditions, as well as interspecies interactions.^146^ The essential oils of various *Ocotea* species also contain minor components such as aldehydes (1.8%), phenylpropenes (1.4%), phenylpropanoids (1.0%), alcohols (0.7%), ketones (0.6%), diterpenes (0.5%), and smaller percentages of aliphatic and aromatic hydrocarbons, carboxylic acids, and esters (Supplementary Tables S6 and S6.1).

#### *Ocotea* spp. Phenylpropanoid Profile

Phenylpropanoids constitute a diverse class of compounds commonly found in plant essential oils, primarily derived from the aromatic amino acid phenylalanine. Safrole, a well-known phenylpropanoid, is notably prevalent across the Lauraceae and was once suggested by Gottlieb as a chemotaxonomic marker for this family due to its widespread occurrence in several species.^8^ Interestingly, to date, the biosynthesis of safrole has only been confirmed in a few *Ocotea* species, such as *O. pretiosa*, *O. odorifera*, *O. cymbarum*, *O. opifera*, and *O. zahamenensis*.^56,146,147^ Notably, safrole is the major component in *O. odorifera* essential oil, where its content can range broadly from 30% to 90%. Despite its prevalence, based on the current data available since Gottlieb’s initial studies, our review suggests that safrole may not serve as a reliable chemotaxonomic marker within the *Ocotea* genus. Nonetheless, it may still hold validity as a marker for other genera within the Lauraceae. This underscores the complexity and variability of the biosynthesis of phenylpropanoids and their potential utility in the chemotaxonomic classification of related plant groups.

### Quantitative Chemical and Pharmacological Overview

The *Ocotea*DB included 984 metabolites reported from *Ocotea* species, spanning 115 species across the genus. These compounds are classified into major chemical groups, including terpenoids (n = 565), of which 491 were identified in essential oils, and 37 were isolated from crude extracts. However, this number of volatile compounds can be considered as approximate due to conventional GC-MS analyses limitations. Followed by lignoids (n = 182), alkaloids (n = 156), flavonoids (n = 61), and other compound classes (n = 57). Regarding species coverage, alkaloids (60 species), lignoids (45 species), flavonoids (20 species), terpenoids (15 species), and other classes (23 species) were reported. A graphical summary of this data is shown (Figure 24), and a full breakdown by species is available in the Supporting Information (Tables S1–9). However, for the evolution of *Ocotea*DB as any genus-tailored chemical database, it should be integrated with geo-referenced data and tissue-specific profiles as more standardized reporting becomes available in the literature.

**Figure 24:**
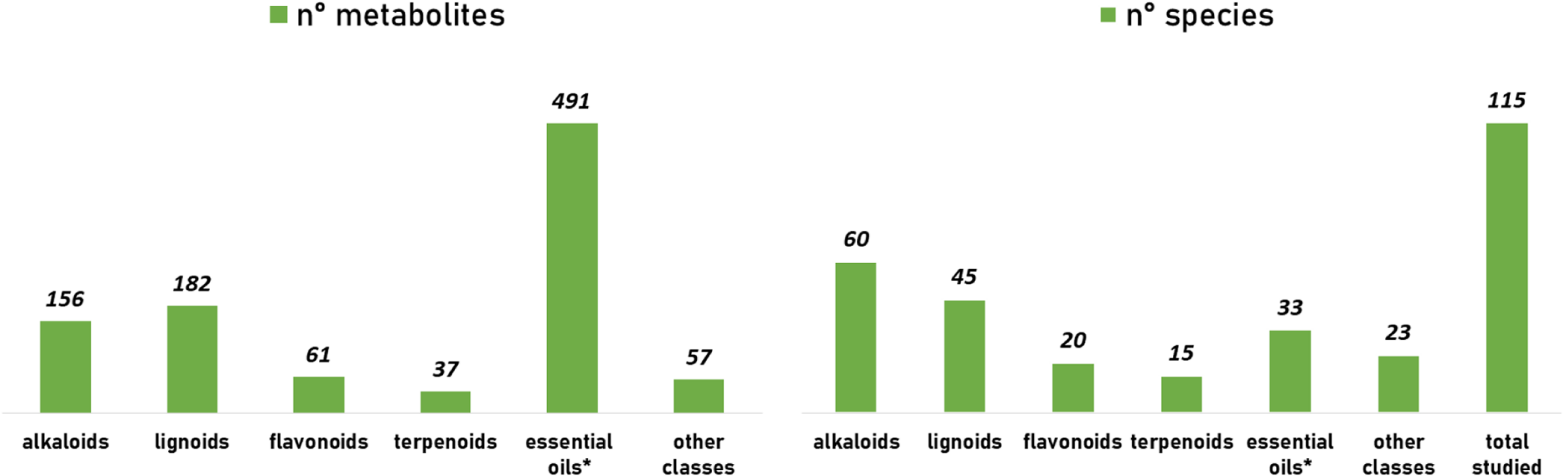
Overview of isolated metabolites (left) and species (right) distribution in *Ocotea* spp. based on data compiled in *Ocotea*DB. *Essential oils included GC-MS reports of mono- and sesquiterpenes.

The biological activity of *Ocotea* metabolites reflects clear structure–activity relationships (SAR) among its major chemical scaffolds. Aporphine alkaloids, the most prevalent class in *Ocotea*, exhibit anti-inflammatory and cytotoxic activities, as shown by compounds like boldine, glaucine, and dicentrine, which act through mechanisms such as COX inhibition, NF-κB suppression, and TRPA1 activation. Benzylisoquinolines observed in *O. odorifera*, *O. lancifolia*, and *O. caudata*, such as **2**, **3** and **4**, have demonstrated anti-inflammatory, antiedematogenic, and anti-HIV effects, attributed to modulation of COX/LOX pathways, butyrylcholinesterase inhibition, and cytokine activity.^4,19,33,34,49,53,56,69^ In parallel, lignans and neolignans such as yangambin, licarin A, and verrucosin, predominantly found in species like *O. cymosa*, *O. duckei*, and *O. macrophylla*, are linked to antiprotozoal, larvicidal, and cytotoxic effects via disruption of protozoal viability, larval development, and mitochondrial integrity, supporting their relevance for neglected tropical diseases.^19,87,115,119^ These correlations between scaffold and dominant bioactivity highlight *Ocotea* as a pharmacologically important genus.

### Recent Advances

Recent research has not only highlighted the cytotoxic and antitumor potential of the metabolome of *Ocotea* species but also demonstrated the significant role that *Ocotea* essential oils play in enhancing the efficacy of antibiotics against multidrug-resistant bacteria. The essential oil extracted from *O. odorifera* and its primary component, safrole, can enhance the activity of macrolide and aminoglycoside antibiotics, such as erythromycin and gentamicin, respectively. This is achieved through the direct inhibition of efflux pumps, which are crucial in modulating bacterial resistance. Consequently, the combination of *O. odorifera* essential oil and safrole with antibiotics leads to improved antibacterial activity and clinically relevant effects against *Staphylococcus aureus*.^177^ Furthermore, alongside their potential as therapeutic agents, recent nanotechnology applications combined with NP present a promising alternative for ecological management. This approach serves as a potential substitute for synthetic antiparasitic drugs and insecticides, which have been linked to significant environmental damage and adverse effects on nontarget organisms, including the development of insect resistance.^174,178,179^ In this context, recently, the potential of a nanoemulsion containing essential oils from *O. pulchella* was evaluated as a control agent in the *Schistosoma mansoni* cycle. This nanoemulsion presented molluscicidal, ovicidal, and cercaricidal activities against the schistosomiasis transmitter *Biomphalaria glabrata*, causing the death of adults and preventing oviposition. The major component found in the studied essential oils is myristicin (methoxy-safrole −29.0%), followed by **36** (17.2%) and **42** (16.6%). Additionally, it showed relevant antiparasitic activity against *S. mansoni*, the infectious agent of schistosomiasis.^180^ Another research demonstrated that an optimized nanoemulsion containing essential oils from *O. indecora* leaves has been developed and tested for its larvicidal properties against *A. aegypti* larvae. The major constituent found in the essential oils was sesquirosefuran (81.4%). *In silico* analysis suggested that the larvicidal property is related to acetylcholinesterase enzyme inhibition. Notably, the nanoemulsion demonstrated no toxicity against the nontarget organism *Apis mellifera*, ensuring safety for pollinator bees. The formulation also showed excellent physical stability under both ambient and refrigerated storage conditions, highlighting its practical viability for vector control applications.^181^

## Conclusion and Perspectives

This review sheds light on the metabolome of the *Ocotea* genus, detailing the principal metabolic pathways that give rise to the bioactive scaffolds of alkaloids, lignoids, flavonoids, and terpenoids. The comprehensive overview of the chemical and biosynthetic characteristics provides a valuable resource for gaining relevant insights and guiding further research targeting *Ocotea* species. It synthesizes data from 115 different *Ocotea* spp. and 984 metabolites, including 156 alkaloids, 182 lignoids, 61 flavonoids, 528 terpenoids (including monoterpenes, sesquiterpenes, diterpenes, triterpenes isolated from crude extracts/identified in essential oils), and 57 metabolites from less-frequent classes. *Ocotea* species represent an important resource for sustainable bioprospecting, especially when studied within frameworks that value biodiversity protection and responsible use of native flora. Integrating chemical exploration with conservation awareness is especially pertinent for Lauraceae, many of which occur in ecologically sensitive or restricted habitats, including several *Ocotea* species. The presented chemical data are suitable for a scaffold library for drug discovery pipelines, in addition to aiding in species identification and chemophenetic studies, providing support for the integration of chemical data with taxonomical investigations in further evolutionary studies of the *Ocotea* genus and Lauraceae. Looking forward, the integration of *Ocotea*DB with emerging genomic and transcriptomic data offers a pathway to fully elucidate the biosynthetic gene clusters responsible for unique bioactive scaffolds present in the genus.

The review also discusses the specific stereochemical differentiation of alkaloids and the presence of common and uncommon NP classes among *Ocotea* species. The structural patterns and metabolites explored herein hold significant potential for the pharmaceutical and medicinal chemistry communities. Promising bioactive scaffolds were detailed, making the *Ocotea* species a valuable source for drug discovery. Future investigations are likely to reveal even more intricate biosynthetic pathways and novel compounds. *Ocotea*DB is an open-access community resource that can facilitate the use of these metabolites in high-throughput virtual screening, particularly in the search for novel anti-inflammatory and antiparasitic leads. Our analysis offers a comprehensive understanding of the *Ocotea* metabolome, providing valuable insights and laying the groundwork for continued advancements in several NP research fields related to the *Ocotea* plant species and its specialized metabolites.

## Supplementary Material



## Notes

Table S1: all *Ocotea* spp. from literature; Table S2: alkaloids; Table S2.1: alkaloids unique-species; Table S3: lignoids; Table S3.1: lignoids unique-species; Table S4: flavonoids; Table S4.1: flavonoids unique-species; Table S5: isolated terpenoids; Table S5.1: isolated terpenoids unique-species; Table S6: essential oils terpenoids; Table S6.1: essential oils terpenoids unique-species; and Table S7: other classes; Table S8: metabolites and Table S9: species; Table S1.1: Katchborian et al. 2024 - Ocotea NP classes hits positive mode; Table S1.2: Katchborian et al. 2024 - Ocotea NP classes hits negative mode (XLSX).
